# Benzydamine hydrochloride for the treatment of sore throat and irritative/inflammatory conditions of the oropharynx: a cross-national survey among pharmacists and general practitioners

**DOI:** 10.1186/s12875-022-01762-3

**Published:** 2022-06-17

**Authors:** Desiderio Passali, Maria Felice Arezzo, Alessandra De Rose, Gianmarco De Simone, Gianluigi Forte, Michalina Jablko-Musial, Ralph Mösges

**Affiliations:** 1 International Federation ORL Societies (IFOS) Executive Board Members, Rome, Italy; 2 Department of Methods and Models for Economics, Territory and Finance, Sapienza University of Rome, Rome, Italy; 3 Farmacie Italiane Group, Rome, Italy; 4 ASL Roma 2, Rome, Italy; 5 Independent Researcher, Katowice, Poland; 6 Institute of Medical Statistics, and Computational Biology (IMSB), Medical Faculty, University at Cologne, Cologne, Germany

**Keywords:** Sore throat, Benzydamine, Oropharynx, General practitioner

## Abstract

**Background:**

Benzydamine for oromucosal use is indicated in the relief of pain and irritation of the mouth and throat. It is an indazole derivative, non-steroidal anti-inflammatory drug, with combined local anesthetic and analgesic properties, and antiseptic activity, marketed under the brand name “Tantum Verde”. The aim of this study was to explore knowledge and prescriptive/advising attitudes among general practitioners (GPs) and pharmacists (PHs) with regard to the topical treatment of sore throat and other irritative/inflammatory conditions of the oropharynx, with a focus on benzydamine. These findings could be important to increase awareness on benzydamine efficacy in sore throat and stomatological conditions, and to reinforce knowledge on the characteristics of benzydamine and its mechanisms of action among healthcare professionals (pediatricians, otolaryngologists, oncologists, etc.).

**Materials and methods:**

An online questionnaire survey was performed among PHs and GPs in four European countries (Italy, Germany, Russia and Poland).

**Results:**

Both GPs and PHs proved to have an excellent knowledge and mastery of the constituents effective against oral symptoms. Among all the principles, benzydamine hydrochloride is the most recognized as certainly suitable for the topical treatment of sore throat symptoms and various inflammatory/irritative conditions of the oral cavity. It is recommended by about 90% of PHs and prescribed by 80% of GPs, mainly to solve the ailments caused by sore throats and stomatitis, especially for its anti-inflammatory, analgesic and anesthetic characteristics. Also in the pediatric field, benzydamine hydrochloride is recommended: among GPs, a high percentage (about 40%) prescribes it like the remedies based on dichlorobenzyl alcohol-sodium benzoate, which are instead more often recommended by PHs (44% against 37%).

**Conclusion:**

Although the public has a lot of confidence in this treatment, GPs and PHs do not recommend/prescribe benzydamine as a first-line treatment of sore throat and other irritative/inflammatory conditions of the oropharynx. To increase the knowledge of benzydamine among these healthcare professionals, it would be important to emphasize its characteristics and the different irritative/inflammatory conditions of the oropharynx in which it can be used.

**Supplementary Information:**

The online version contains supplementary material available at 10.1186/s12875-022-01762-3.

## Introduction

Benzydamine is an indazole derivative, non-steroidal anti-inflammatory drug, with combined local anesthetic and analgesic properties, and antiseptic activity. It is an over-the-counter drug in Europe (except in Turkey) marketed under the brand name “Tantum Verde” [1].

In addition to its anti-inflammatory activity, benzydamine shows local an analgesic/anesthetic effects that in the topical route can be fully exploited and turned into competitive advantages over Non-Steroidal Anti-Inflammatory Drugs (NSAIDs) [2, 3]. In fact, its elective therapeutic use is the topical control of acute inflammation and pain. Benzydamine has its target indication in the symptomatic treatment of pain and irritative/inflammatory conditions of the oropharynx (gingivitis, stomatitis, pharyngitis), even when due to dental therapy.

Local application of benzydamine produces higher concentrations in the inflamed area than in the blood, as shown in studies performed in animals and humans [4, 5]. The ability of benzydamine to concentrate in the inflamed tissues, with low systemic exposure, is a clear advantage that limits potential systemic side effects [6].

The three main activities of benzydamine, anti-inflammatory, local analgesic, and anesthetic, all contribute to a highly specific and targeted efficacy, reducing symptoms related to a local inflammatory state.

Benzydamine hydrochloride has been studied and found to be effective for treating oral mucositis and oral ulcers, as well as for the prevention of post-operative sore throat following endotracheal intubation [7, 8]. Moreover, in some countries, it is also indicated for the symptomatic treatment of oromucosal inflammation, including oral mucositis due to radiotherapy + /chemotherapy in patients with Head and Neck Cancer (HNC) [9, 10].

Understanding the prescribing practice and attitudes among general practitioners (GPs) and community pharmacists (PHs) would help ascertain the degree of awareness of different active ingredients, including benzydamine hydrochloride, as topical treatments for irritative/inflammatory conditions of the oropharynx. These findings could be important to increase awareness on benzydamine efficacy in sore throat and stomatological conditions, and to reinforce knowledge on the characteristics of benzydamine and its mechanisms of action among healthcare professionals (pediatricians, otolaryngologists, oncologists, etc.).

Therefore, a survey among GPs and PHs in different European countries was performed with the aim of exploring knowledge, attitudes and prescriptive/advising behaviour of the respective study populations with regard to the topical treatment of sore throat and other irritative/inflammatory conditions of the oropharynx, with a focus on benzydamine.

## Materials and methods

### Study design

This study was carried out through an online questionnaire survey among PHs and GPs in four European countries (Italy, Germany, Russia and Poland) participating in the project. The questionnaire was sent out via e-mail by country-specific research marketing companies operating in the pharmaceutical sector and quality-certified (ISO 20252). The study was conducted between June and July 2021. In each country, 150 questionnaires were sent to GPs and 150 to community PHs, and the top 100 in each category were collected, amounting to a total of 802* completed questionnaires.

* One more questionnaire was collected from PHs in Poland and one more questionnaire was collected from GPs in Russia.

### The questionnaire

The questionnaire was designed by an interdisciplinary research team consisting of experienced GPs, otolaryngologists, a social scientist, and a statistician. The aforementioned team has a long standing experience in both practicing medicine and in research. An initial draft of the survey was reviewed by a general practitioner external to the research team to ensure it was easy to understand and could be completed in a few minutes. Two slightly different versions, according to the specific skills and tasks of the two target populations – community PHs and GPs—have been developed and then translated into the languages of the four countries involved. The questionnaire in English can be found in the appendix (see Additional file 1). It dealt with a total of 17 and 15 items to the GPs and PHs, respectively.

The main topics of the questionnaire can be summarized as follows. The first question regarded what the respondent – based on own knowledge – thinks about the usability of different active ingredients, including benzydamine hydrochloride, for the mouth and throat irritative symptoms. The next four items asked which of the active ingredients has been generally prescribed (if GP) or recommended (if PH), and what formulation, to adults and children, respectively. Then, focusing on those GPs and PHs who declared to prescribe and/or recommend benzydamine hydrochloride, a number of items inquired about which symptoms and mechanisms of action (anesthetic, analgesic, anti-inflammatory, antiseptic) it is mainly recommended for. PHs were asked to give an evaluation of the proportion of their customers acquiring the product based on benzydamine hydrochloride by themselves, that is an indicator of self-medication. The last items of the questionnaire collected information on demographic characteristics and the number of patients/customers usually followed by the respondent GP/PH.

### Statistical analysis

All results are based on point estimations and hypothesis testing. Separately for GPs and PHs, frequencies for all data collected were reported. For presentation purposes, categorical data were presented as percentages and continuous data as means and standard deviations (SDs). Differences between categories of interest and countries were analyzed using chi-square tests and t tests. A value of *P* < 0.05 was considered statistically significant. All analyses were performed using R, a programming language and free software environment for statistical computing and graphics supported by the R Core Team and the R Foundation for Statistical Computing.

## Results

### Characteristics of respondents

Participant demographic details are shown in Table 1.

**Table 1:** Characteristics of GPs and PHs participating in the study

	**General practitioners**				**Pharmacists**			
	Italy	Germany	Russia	Poland	Italy	Germany	Russia	Poland
Gender								
% Females	41	46	50	35	44	50	49	39
Age Mean (SD)	51.0 (9.3)	56.8 (8.9)	49.9 (11.6)	52.1 (7.2)	44.0 (10.5)	53.9 (9.3)	48.6 (11.6)	52.2 (7.3)
N. of Patients/Customers Mean (SD)	1097.78 (252.6)	1162.85 (222.3)	1004.16 (289.8)	1219.69 (299.7)	533.5 (163.4)	539.9 (177.3)	539.2 (212.3)	517.9 (251.9)
N. of Respondents	100	100	101	100	100	100	100	101

Among both pharmacists and general practitioners, men slightly outnumber women, and it is interesting to note that there are differences between countries: in Poland and Italy, the gender difference is more pronounced, especially among GPs. The overall mean age of the pharmacists is lower than general practitioners’: 49.6 (SD = 10.48) for PHs *vs* 52.5 (SD = 9.72) for GPs. On customers/patients base, approximately 90% of PHs have more than 300 customers with a mean of 533.3 (SD = 201.3), while for GPs the average number of patients is 1120.3 (SD = 278.6).

### Benzydamine hydrochloride (Tantum Verde) and the other treatments

The vast majority of respondents, both GPs and PHs, reported the usability of all six active ingredients listed in the questionnaire as topical treatments for sore throat and inflammatory/irritative conditions of the mouth (Table 2).

**Table 2:** Reported usability in the survey of active ingredients for topical medication of sore throat and inflammatory/irritative conditions of the mouth (i.e. gingivitis or stomatitis)

**GPs**						
Values	Ketoprofen	Flurbiprofen	Ambroxol Chlorydrate	Dichlorobenzyl alcohol-Sodium Benzoate	Benzydamine Hydrochloride	Natural extracts
Yes	75.1	80.3	85.5	85.3	92.3	62.6
No	20.2	17.7	13.0	12.7	6.5	26.9
Don’t know	4.7	2.0	1.5	2.0	1.2	10.5
**PHs**						
Values	Ketoprofen	Flurbiprofen	Ambroxol Chlorydrate	Dichlorobenzyl alcohol-Sodium Benzoate	Benzydamine Hydrochloride	Natural extracts
Yes	76.3	81.8	89.5	91.8	95.3	73.1
No	20.2	15.7	8.7	7.2	3.7	18.5
Don't know	3.5	2.5	1.7	1.0	1.0	8.5

Values inside the table are percentages

Respondents showed an excellent knowledge of treatments for oral pain conditions in general and of benzydamine hydrochloride, in particular. In fact, among the six possible constituents listed in the questionnaire, benzydamine hydrochloride was the most frequently recognized effective treatment for sore throat and pain conditions in the mouth, with 92.3% of GPs and 95.3% of PHs declaring it as usable in such situations.

GPs and PHs concur in the usability of the active ingredients and their approach is similar for the other topical medications; the only remarkable difference is between natural extract and all the other treatments with an average difference of about 14% for PHs and 21% for GPs.

Regarding the usability of benzydamine, no differences between countries were found (Table 3): chi-square test state the equality of percentages of “yes” answers among the four countries analyzed for GPs (*p*-value = 0.758) and for PHs (*p*-value = 0.868). Looking at the differences between GPs and PHs within each country, the only statically significant result is in Italy (*p*-value = 0.0035) where PHs state usability of benzydamine in higher percentage (100% *vs* 89.10%).

**Table 3:** Reported usability in the survey of benzydamine for topical medication of sore throat and inflammatory/irritative conditions of the mouth (i.e. gingivitis or stomatitis) by country

Value	Country	GPs	PHs
Yes	DE	87.00	89.00
No	DE	10.00	9.00
Don't know	DE	3.00	2.00
Yes	ITA	89.10	100.00
No	ITA	9.00	0.00
Don't know	ITA	1.00	0.00
Yes	PL	92.00	94.10
No	PL	6.90	4.00
Don't know	PL	1.00	2.00
Yes	RUS	100.00	98.00
No	RUS	0.00	2.00
Don't know	RUS	0.00	0.00

Values inside the table are percentages

Regarding prescribing, most respondents answered "yes" with a similar approach across treatments and between GPs and PHs, except for natural extracts (Table 4).

**Table 4:** Percentages of GPs and PHs that prescribe/recommend the active ingredients for topical treatment of symptoms of sore throat and various inflammatory/irritative conditions of the oral cavity such as gingivitis and stomatitis

**GPs**						
Values	Ketoprofen	Flurbiprofen	Ambroxol Chlorydrate	Dichlorobenzyl alcohol-Sodium Benzoate	Benzydamine Hydrochloride	Natural extracts
Total	69.80	76.10	81.30	83.50	80.80	49.10
DE	70.00	71.00	74.00	81.00	73.00	40.00
ITA	63.00	67.00	72.00	72.00	89.00	46.00
PL	69.00	86.00	91.00	94.00	72.00	42.00
RUS	78.00	81.00	89.00	88.00	90.00	69.00
**PHs**						
Values	Ketoprofen	Flurbiprofen	Ambroxol Chlorydrate	Dichlorobenzyl alcohol-Sodium Benzoate	Benzydamine Hydrochloride	Natural extracts
Total	72.10	81.30	89.80	90.30	89.30	57.40
DE	54.00	64.00	80.00	83.00	81.00	46.00
ITA	95.00	98.00	94.10	96.00	98.00	70.30
PL	70.00	87.00	93.00	93.00	86.00	49.00
RUS	68.30	75.20	91.10	88.10	91.10	63.40

Values inside the table are percentages

The three constituents most recommended by PHs for the topical treatment of sore throat and other oral conditions are dichlorobenzyl alcohol-sodium benzoate (90.3%), ambroxolo hydrochloride (89.8%), and benzydamine hydrochloride (89.3%). Benzydamine hydrochloride is recommended by a very high percentage of GPs (80.8%) in the topical treatment of the oral cavity and is surpassed by dichlorobenzyl alcohol-sodium benzoate, which is prescribed by a slightly higher percentage of GPs (83.5%). Regarding approach to benzydamine between countries (Table 4), no statistical difference was found either for PHs (*p*-value = 0.5678) or for GPs (*p*-value = 0.3105). Within countries, there is a statistically significant difference between PHs and GPs only in Italy (*p*-value = 0.00737) and in Poland (*p*-value = 0.0357).

End users appear very familiar with benzydamine hydrochloride, as evidenced by the fact that on average 40.03% of people require it as a self-medication (Table 5).

**Table 5:** Customer management of purchases of benzydamine hydrochloride (average percentage according to pharmacists)

	Self-management	On medical advice	On pediatrician advice	On pharmacist advice
Total Mean	40.03	24.24	18.98	16.75
DE mean	36.85	25.25	16.35	21.55
IT mean	40.15	25.85	22.65	11.35
PL mean	40.89	22.40	16.47	20.25
RUS mean	42.2	23.5	20.48	13.82

Values inside the table are percentages

### The mechanisms of action and symptoms for which benzydamine hydrochloride is recommended

Based on pharmacists’ experience, the first two symptoms that prompt clients to seek advice are ache (14.57%) and cough (11.65%), followed by redness of the throat (9.37%), itchy throat and symptoms due to extraction therapy (Table 6).

**Table 6:** Average percentage of clients who seek for the advice of pharmacists by throat and oral symptoms

	*All countries (mean)*	*Germany (Mean)*	*Italy (Mean)*	*Poland (Mean)*	*Russia (Mean)*
Ache	14.57	8.67	22.2	12.03	15.41
Itchy throat	8.87	5.49	14.61	5.05	10.37
Difficulty in swallowing	4.91	5.69	3.24	4.90	5.79
Dry throat	3.95	4.06	3.05	4.89	3.80
Mouth throat burning	4.96	7.26	1.88	6.19	4.51
Redness of the throat	9.37	7.51	13.16	8.43	8.39
Tonsillitis	7.88	10.19	5.32	8.74	7.24
Distorted voice	2.90	3.08	1.32	3.45	3.76
Cough	11.65	7.78	15.27	11.90	11.64
Ulcerative lesions or canker sores	6.06	10.02	0.80	6.57	6.86
Halitosis	4.84	6.74	2.27	4.26	6.10
Temperature	4.67	4.14	5.05	6.22	3.27
Symptoms due to extraction therapy	8.18	6.86	8.45	10.05	7.33

Values inside the table are percentages

Clients behave very differently depending on the country (Table 6): for example, in Germany only an average of 8% of customers ask for advice if they have ache, while in Russia this percentage is almost double and in Italy almost triple. Even for cough, in Germany only 7.78% of customers ask for advice, while in Italy this percentage is double.

The vast majority of GPs (71.8%) and PHs (76.8%) recommend benzydamine hydrochloride to their patients/customers, with no significant difference in approach between GPs and PHs (*p*-value = 0.1246), as these proportions are not statistically different.

Benzydamine hydrochloride is prescribed for all four of its properties by both PHs and GPs who declared to prescribe/recommend benzydamine hydrochloride (Table 7). The most appreciated characteristic of benzydamine hydrochloride is its anti-septic features (71.60% for GPs and 74.10% for PHs), followed by the anti-inflammatory for PHs (68.20%) and anesthetic for GPs (60%).

**Table 7:** Percentage of GPs and PHs that top rank benzydamine hydrochloride by its characteristics

**GPs**				
Values	*Anesthetics*	*Analgesics*	*Anti-inflammatory*	*Antiseptics*
4 or 5	60.00	55.50	58.70	71.60
**PHs**				
Values	*Anesthetics*	*Analgesics*	*Anti-inflammatory*	*Antiseptics*
4 or 5	64.60	62.90	68.20	74.10

Question administrated only to those who declared to prescribe BZD: 287 GPs and 307 PHs, respectively

Values inside the table are percentages out of these sub-totals

Sore throat is by far the pathological condition for which GPs (27.52%) and PHs (32.89%) more often recommend the use of benzydamine hydrochloride, followed by gingivitis (20.28% for PHs and 19.25% for GPs) (Table 8).

**Table 8:** Percentage of times benzydamine hydrochloride is recommended for each pathological condition

	**GPs**						**PHs**				
	All Countries (mean)	Germany (mean)	Italy (Mean)	Poland (Mean)	Russia (Mean)		All Countries (mean)	Germany (mean)	Italy (Mean)	Poland (Mean)	Russia (Mean)
Gingivitis	19.25	18.85	20.07	15.97	21.8		20.28	22.01	20.46	20.06	18.53
Stomatitis	16.73	20.18	14.94	17.76	14.33		16.23	18.54	10.61	18.21	16.86
Conservative dental therapy	16.23	18.7	18.83	13.81	13.3		13.79	13.29	10.81	14.36	16.4
Extractive dental therapy	15.84	13.47	17.01	17.99	14.96		12.78	10.67	10.79	15.51	14.05
Sore throat	27.52	25	26.69	33.81	25.07		32.89	33.05	42.71	30.51	26.28
Other	4.43	3.8	2.47	0.67	10.55		4.04	2.44	4.61	1.35	7.89

Question administrated only to those who declared to prescribe BZD: 287 GPs and 307 PHs, respectively

Values inside the table are percentages out of these sub-totals

Essentially, there is the same approach at country level, with slight differences for GPs and PHs in Italy, where the former recommend benzydamine about 26.69% of the time, while the latter recommend it on average about 42.71% of the time.

Ache and cough are the symptoms for which the PHs more often suggest the use of benzydamine hydrochloride. Unlike PHs, the symptoms for which GPs most often give indications to use benzydamine are those related to extraction therapy (Table 9).

**Table 9:** Percentage of GPs and PHs who recommend benzydamine hydrochloride by client-reported symptoms

	GPs (mean)	PHs (mean)
Ache	10.79	12.68
Itchy and itchy throat	7.69	8.05
Difficulty in swallowing	5.70	5.30
Dry throat	3.92	3.81
Burning of the mouth	8.29	5.24
Redness of the throat	8.58	9.23
Distorted voice	3.14	2.75
Cough	10.74	10.43
Ulcerative lesions or canker sores	11.34	8.23
Halitosis	4.98	4.13
Temperature	1.60	2.51
All symptoms due to extraction therapy	14.38	9.62

Question administrated only to those who declared to prescribe BZD: 287 GPs and 307 PHs, respectively

Values inside the table are percentages out of these sub-totals

There are significant differences across symptoms (*p*-value < 0.001), but not between GPs and PHs (all *p*-values > 0.05).

### The use of benzydamine hydrochloride in pediatrics

While natural extracts are much less recommended, both active ingredients, benzydamine and dichlorobenzyl alcohol-sodium benzoate, are prescribed/recommended for the treatment of sore throat in the pediatric population by GPs (39.7 and 40.6%) and PHs (36.9 and 44.4%) in a similar proportion (Table 10), with no statistical difference between treatments and between GPs and PHs (*p*-values > 0.05).

**Table 10:** Preference distribution for the active ingredients prescribed/recommended for the topical treatment of sore throat symptoms in children (order of preference: 1 = most preferred, 3 = least preferred)

		**GPs**			**PHs**		
Preference	Country	Benzydamine Hydrochloride	Dichlorobenzyl Alcohol, Sodium Benzoate	Natural extracts	Benzydamine Hydrochloride	Dichlorobenzyl Alcohol, Sodium Benzoate	Natural extracts
1 Highest	All	39.70	40.60	19.50	36.90	44.40	18.70
2	All	40.10	44.10	15.50	39.20	48.10	12.70
3 Lowest	All	20.20	15.20	65.00	23.90	7.50	68.60
1 Highest	DE	38.00	40.00	21.00	39.00	43.00	18.00
2	DE	46.00	47.00	6.00	41.00	48.00	11.00
3 Lowest	DE	16.00	12.00	70.00	19.80	8.90	70.30
1 Highest	IT	60.40	33.00	6.00	42.00	46.00	12.00
2	IT	30.00	45.00	25.00	36.00	51.00	13.00
3 Lowest	IT	9.00	22.00	69.00	22.00	3.00	75.00
1 Highest	PL	28.00	45.00	27.00	32.70	51.50	15.80
2	PL	47.50	43.00	9.00	51.00	43.00	7.00
3 Lowest	PL	24.00	12.00	64.00	17.00	6.00	78.00
1 Highest	RUS	32.00	45.00	24.00	34.00	37.00	29.00
2	RUS	37.00	42.00	22.00	28.70	50.50	19.80
3 Lowest	RUS	31.70	14.00	55.00	37.00	12.00	51.00

Values inside the table are percentages

Looking at countries in more detail, there are different approaches among countries and between GPs and PHs, and of note, benzydamine is by far the most recommended active ingredients by Italian GPs (60.4%) for topical treatment of sore throat symptoms in children (Table 10).

The three formulations, spray, hard candy and soft tub, are equally liked by GPs and by PHs. Regarding the preferred formulation, the spray treatment mode is the most recommended by both GPs (38.4%) and PHs (35.2%) for treating children (Table 11).

**Table 11:** Preference distribution for the formulations prescribed/recommended for the treatment of sore throat symptoms in children (order of preference: 1 = most preferred, 3 = least preferred)

		**GPs**			**PHs**		
Preference	Country	*Spray*	*Hard candy*	*Soft tabs*	*Spray*	*Hard candy*	*Soft tabs*
1 Highest	All	38.40	26.60	34.90	35.20	31.70	33.20
2	All	37.70	40.70	21.60	27.40	41.40	31.20
3 Lowest	All	23.90	32.70	43.50	37.40	26.90	35.70
1 Highest	DE	50.00	20.00	27.00	54.00	29.00	17.00
2	DE	34.00	45.00	18.00	28.00	46.00	26.00
3 Lowest	DE	13.00	32.00	52.00	17.80	24.80	56.40
1 Highest	IT	43.00	23.00	34.00	24.00	34.00	42.00
2	IT	40.00	42.00	18.00	24.00	34.00	42.00
3 Lowest	IT	17.00	35.00	48.00	52.00	32.00	16.00
1 Highest	PL	32.00	25.00	43.00	29.70	31.70	38.60
2	PL	44.00	36.00	20.00	34.00	43.00	24.00
3 Lowest	PL	24.00	39.00	37.00	37.00	26.00	38.00
1 Highest	RUS	28.00	38.00	35.00	33.00	32.00	35.00
2	RUS	32.00	39.00	30.00	23.80	42.60	32.70
3 Lowest	RUS	41.00	24.00	36.00	43.00	25.00	32.00

Values inside the table are percentages

Some differences emerge when looking more closely at the countries: for example, in Germany the spray is the preferred treatment, whereas in Poland both GPs and PHs prefer soft tablets over other formulations. In Italy, it is interesting to note a different approach between GPs and PHs: in fact, Italian GPs prefer spray, while PHs prefer soft tablets (Table 11).

## Discussion

### Comparison of benzydamine hydrochloride with other drugs

In this survey both GPs and PHs proved to have an excellent knowledge and mastery of the constituents effective against oral symptoms. Among all the principles, benzydamine hydrochloride is the most recognized as certainly suitable for the topical treatment of sore throat symptoms and various inflammatory/irritative conditions of the oral cavity such as gingivitis and stomatitis, with more than 92% of the interviewed GPs and more than 95% of the respondent PHs declaring it as usable in such situations.

In addition, GPs and PHs agree on the usability of other active ingredients, recognizing Non Steroidal Anti-Inflammatory Drugs (NSAIDs) such as flurbiprofen and ketoprofen, the mucolytic agent ambroxol, and the active ingredients dichlorobenzyl alcohol and sodium benzoate with local antiseptic action of the oropharyngeal cavity as effective medications for sore throats and inflammatory/irritative conditions of the mouth. Moreover, natural remedies could also be helpful with products capable of exerting an anti-inflammatory action towards the throat.

Dichlorobenzyl Alcohol-Sodium Benzoate is the most widely prescribed by GPs and recommended by PHs for the topical treatment of sore throat and other oral conditions, followed by ambroxol hydrochloride and benzydamine hydrochloride in a slightly lower percentage.

As for benzydamine hydrochloride, it is recommended by about 90% of PHs and prescribed by 80% of GPs, with no significant differences between countries. There are no data in the literature to support these preferred recommendations. These findings represent clinical practice and real-life data from the countries that participated in the survey.

Moreover, end users appear very familiar with benzydamine hydrochloride, as evidenced by the fact that on average 40% of people require it as a self-medication. This is confirmed across nations, so regardless of the country most customers ask for the benzydamine on a self-management basis. This attitude can be explained by the fact that benzydamine is an over-the-counter (OTC) drug and does not require a prescription from a healthcare professional. Moreover, the high rate of self-management shows that patients/customers are confident with benzydamine and also very satisfied with the therapeutic outcome.

The lower rate of pharmacist advice for benzydamine compared to medical advice (17% *vs* 24%) may be explained by the fact that doctors and physician cover different roles. Generally customers prefer to follow their doctor's prescription, while those who seek advice from the pharmacist have already chosen the self-management route. Pharmacists should offer reassurance and advice on appropriate treatment to increase the chances of optimum symptomatic relief and satisfaction [11]. In addition, the pharmacist can leverage on a plethora of treatment for sore throat and it is worth considering that treatment decisions are not only influenced by medical factors; non-medical factors such as cultural issues or patient expectations and pressures also influence the prescribing approach [12].

### The mechanisms of action and symptoms for which Benzydamine Hydrochloride is recommended

The first two symptoms that prompt clients to seek advice to the pharmacist are ache and cough, followed by redness of the throat, itchy throat and symptoms due to extraction therapy. However, clients behave very differently depending on the country: this different attitude reflects the cultural and social characteristics of the country and can also be influenced by a different healthcare system.

The great majority of PHs (77%) recommend benzydamine hydrochloride to their customers and the 72% of the interviewed GPs usually prescribe it, mainly to solve the disorders caused by sore throat, followed by gingivitis and stomatitis.

It is important to consider that while the etiology of gingivitis or stomatitis is clearly defined, sore throat is an inflammatory condition characterized by pain, redness, heat and swelling that has different causes [13]. Sore throat is a term often used to describe pharyngitis, tonsillitis and laryngitis that occur for a short period of time, which result from inflammation of the upper respiratory tract. Tonsillitis is inflammation due to infection of the tonsils, whereas pharyngitis is inflammation of the oropharynx only but, in practice, the distinction between the two can be unclear and they can occur simultaneously. Laryngitis is used when there is hoarseness with soreness lower down in the throat [14].

The non-infective causes of sore throat are usually due to environmental variations, such as temperature changes, low humidity, second hand smoking, air pollution and a reaction to allergens [15]. Only a small proportion of sore throat cases are caused by bacterial infection, such as group A beta-hemolytic streptococcus, and up to 80% of sore throats are caused by viruses, such as influenza A, respiratory syncytial virus, severe acute respiratory syndrome corona virus, and rhinovirus [16, 17]. Therefore, antibiotics are ineffective in most cases and are not recommended as the first line of treatment for acute sore throat in the EU [18, 19]. However, irrespective of causative pathogen, there is a need for symptom relief. Most patients with acute sore throat present with symptoms which include pain on swallowing, headache and cough, and flu-like symptoms.

This survey shows that ache and cough are the symptoms for which the PHs more often suggest the use of benzydamine hydrochloride (12,68% and 10,43%, respectively). Differently from the PHs, the symptom for which GPs most often give indications to use benzydamine are those related to extraction therapy (14,38%). However, the symptoms for which GPs and PHs recommend benzydamine vary depending on the country, probably reflecting a different clinical practice experience.

Benzydamine hydrochloride is prescribed/recommend for all four of its properties (anti-inflammatory, analgesic, anesthetic, and antiseptic) by both GPs and PHs. The most appreciated characteristics of benzydamine hydrochloride are its anti-inflammatory features, followed by the anesthetic ones for both GPs and PHs.

In fact, the pharmacological activity of benzydamine is mainly related to the inhibition of proinflammatory cytokine synthesis, although it also shows anesthetic activity by modulating neuronal excitability.

Although benzydamine is a nonsteroidal anti-inflammatory agent, it possesses a different mechanism of action that distinguishes it from conventional NSAIDs. Unlike NSAIDs that act by inhibiting prostaglandin synthesis, the anti-inflammatory activity of benzydamine has been related to its ability to inhibit the release of pro-inflammatory cytokines (TNFa, IL-1β, and MCP-1), without affecting other inflammatory cytokines (IL-6, IL-8), and, importantly, anti-inflammatory cytokines (IL-10, IL-1ra) [20].

However, it also exerts other activities that may contribute to the modulation of the inflammatory status, i.e. reduction of vascular permeability produced by histamine, acetylcholine, serotonin and epinephrine, inhibition of platelet aggregation, thrombus formation, degranulation of human polymorphonuclear leukocytes, and migration of human monocytes.

The local anesthetic properties of benzydamine are most likely due to its structural features. Unlike other NSAIDs, it does not associate with steroid structure and it shares with local anesthetics an aromatic (hydrophobic) ring structure linked to a basic tertiary amine group (hydrophilic) by a short alkyl chain [21]. Therefore, benzydamine like local anesthetics, reversibly blocks nerve conduction when applied topically in appropriate concentrations.

The results of a study showed that benzydamine blocks voltage-gated Na + currents in Dorsal Root Ganglion (DRG) neurons, and suggest that it can be a modulator of nociceptor excitability [22]. Indeed, benzydamine attenuates the local transmission of the pain stimulus by preventing the generation and propagation of action potentials through the blockade of Na_v_ channels expressed in sensory neurons. In a clinical trial performed on 87 healthy subjects, benzydamine, applied topically to normal mucosa for 60 s, was found to exert a remarkable anesthetic activity superior to control (cetylpyridinium hydrochloride 0.025%) and placebo mouthwashes, also showing a long lasting effect (more than 90 min). The local anesthetic activity of benzydamine has been shown to be extremely useful in the treatment of painful conditions of the mouth and throat, primarily due to rapid pain relief [2].

### The use of benzydamine hydrochloride in pediatrics

Also in the pediatric field, benzydamine hydrochloride is indicated for the topical treatment of sore throat symptoms. Among GPs, a high proportion (about 40%) prescribe benzydamine in a similar proportion to other remedies based on dichlorobenzyl alcohol, which is slightly preferred by PHs instead (44% *vs* 37%). Looking at countries in more detail, except for Italian physicians who prescribe mostly benzydamine (60%), both GPs and PHs prefer dichlorobenzyl alcohol for topical treatment of sore throat symptoms in children.

Dichlorobenzyl alcohol (DCBA) is a mild antiseptic used as an active ingredient in several marketed OTC throat lozenges, typically in combination with Amylmetacresol (AMC), another antiseptic, for the symptomatic relief of mouth and throat infections, and acute sore throat due to upper respiratory tract infections (URTIs) [23, 24].

Lozenges containing AMC/DCBA have been reported in several clinical trials in adults and have proven to produce significantly greater improvement for symptomatic and pain relief, such as difficulty swallowing and throat numbness, and a reduction in the severity of throat soreness in patients with URTIs [23, 25]. AMC/DCBA throat lozenges have been shown to be safe and efficient in relieving of acute sore throat symptoms, and it produces an immediate symptomatic relief [26, 27]. Moreover, *in-vitro* evidence has demonstrated the virucidal effect of lozenges containing AMC and DCBA on a number of viruses associated with the common cold such as RSV, influenza A virus, and a member of the coronavirus family, SARS-CoV; a reduction in viral load is believed to have benefits in reducing the symptoms [28].

The characteristics of this active ingredient, at the country level, might justify the greater preference for dichlorobenzyl alcohol over benzydamine. However, there are no clinical studies that have compared these two active ingredients for the topical treatment of sore throat symptoms.

Although in a smaller percentage, natural extracts are prescribed/recommended by GPs and PHs, respectively. This trend can be explained by the influence of parents who sometimes prefer to avoid "chemical" drugs and are more prone to give their children natural products. This is an obstacle that should be overcome by convincing these parents that there are effective and safe pharmacologic alternatives for sore throat symptoms in children.

The three formulations, spray, hard candy and soft tub, are equally liked by physicians and by pharmacists. Regarding the preferred formulation, the spray treatment mode is the most recommended by both GPs (38.4%) and PHs (35.2%) for treating children. However, the choice of drug formulation in pediatrics also depends on the age of the children and the preferences of the parents, and on the safety profile. Children, for example, prefer the sweet effect of a candy, while parents the convenience of a spray because they can control the mode of application. Moreover, the palatability of medications is an important factor in determining medication adherence and completion of drug therapy in young children, and should be an important part in the choice of pediatric formulations [29].

There are clear differences between the topical delivery systems in the onset of action and the amount of active ingredients present in the mouth and throat [30]. Advantages of a spray include delivery of a full dose immediately at the site of pain and inflammation, whereas lozenges slowly dissolve to release the active ingredients directly onto the irritated mucosal tissues [31].

In conclusion, benzydamine can be considered an effective treatment in children and can be administered as a spray in localized forms such as in the treatment of inflamed gum in dental eruption or as soft tab for a more generalized sore throat.

### Strengths and limitations of the study

In this survey, benzydamine hydrochloride is the most recognized as certainly suitable for the topical treatment of sore throat symptoms and various inflammatory/irritative conditions of the oral cavity. It is recommended by about 90% of PHs and prescribed by 80% of GPs mainly to resolve complaints caused by sore throat and stomatitis, with no significant differences between countries. These results are interesting because no recommendations on the use of benzydamine for the symptomatic treatment of sore throat appear in most European guidelines on the management of acute sore throat [13, 32–35]. Therefore, these results represent clinical practice and real-life data from the countries that participated in the survey, providing an updated picture of benzydamine use compared with other drugs in adult and pediatric populations.

However, there were several limitations to this study.

The first limitation is that the active ingredients compared in the study, though OTC drugs and freely available, may have slightly different therapeutic indications in each country. This may explain the different prescription rates in the countries involved in the survey. In particular, although benzydamine for oromucosal use is indicated in the relief of pain and irritation of the mouth and throat, the approved indication differs slightly among countries.

The second is that we used a self-reported survey so it is possible that GPs and PHs answered the questions in a way that reflected professional desirability rather than their practices.

Third, the different healthcare systems in each country involved in the study should be considered to explain some differences in the results; for example, in Germany, patients do not have to go to a general practitioner and then be referred to a specialist, and pharmacies are still owned by individual pharmacists. Therefore, the way to reinforce knowledge of benzydamine should differ depending on the market and healthcare system in the individual country.

## Conclusions

This study suggests that interventions to improve knowledge of GPs and PHs about topical treatment of sore throat and other irritative/inflammatory conditions of the oropharynx, particularly benzydamine, are warranted.Although customers/patients have a lot of confidence in this treatment, these specialists do not recommend/prescribe benzydamine as a first-line treatment. To fill this gap it should be fundamental to increase knowledge of benzydamine among these healthcare professionals, emphasizing its characteristics and the different irritative/inflammatory conditions of the oropharynx in which it can be used. In particular, it would be useful to improve knowledge of among pharmacists, who are in contact with the public, about the efficacy of benzydamine and its mechanisms of action, so that they can proactively recommend it in the treatment of sore throat and thus reduce the burden on the healthcare system and the workload of general practitioners.

Several projects are currently underway to collect new data on the use of benzydamine in the treatment of sore throat and to thoroughly investigate its mechanisms of action. Of note, a recently completed phase IV study (BePaiR study) -results not yet published- evaluated the efficacy and speed of relief provided by a single application of benzydamine hydrochloride (0.30% spray or lozenges) in patients with acute sore throat (NCT04941976).

In conclusion, the results of this study are important because they represent clinical practice and real-life data from the countries that participated in the survey, providing an updated picture of benzydamine use in the treatment of inflammatory and irritative condition of the throat and mouth compared with other drugs in adult and pediatric populations.

## Supplementary Information

**Additional file 1.**

## Data Availability

The data that support the findings of this study are available as supplementary materials.
