# Supplementary material for: Benzydamine hydrochloride for the treatment of sore throat and irritative/inflammatory conditions of the oropharynx: a cross-national survey among pharmacists and general practitioners
Source: BMC Prim Care. 2022 Jun 17;23:154. doi: 10.1186/s12875-022-01762-3 (PMC9205545; doi:10.1186/s12875-022-01762-3)
Supplement: Supplementary file 1 — Additional file 1. [file 12875_2022_1762_MOESM1_ESM.zip › Appendix 1 - Survey.pdf]

Appendix 1

Questionnaire survey for Pharmacists and General Practitioners in 4 European countries (Italy, Germany, Russia and Poland)

Survey for Pharmacists

D1. Based on your knowledge, are the following constituents of a drug are usable for the topical medication of sore throat and inflammatory/irritative conditions of the mouth such as gingivitis or stomatitis?

Ketoprofen

Flurbiprofen

Ambroxol Chlorydrate

Dichlorobenzyl alcohol - Sodium Benzoate

Benzydamine Hydrochloride

Natural extracts[1]

D2. Generally speaking, which constituents do you advise for the topical treatment of sore throat and inflammatory/irritative conditions of the mouth such as gingivitis or stomatitis?

Ketoprofen

Flurbiprofen

Ambroxol Chlorydrate

Dichlorobenzyl alcohol - Sodium Benzoate

Benzydamine Hydrochloride

Natural extracts[1]

D3. Which of the following formulations do you recommend for the treatment of sore throat symptoms in adults? Assign an order of preference to each (1 highest, 4 lowest preference)

Spray

Hard candy

Mouthwash

Soft tab

D4. Which of the following constituents do you recommend for the topical treatment of sore throat symptoms in children? Assign an order of preference to each (1 highest, 3 lowest preference)

Benzydamine Hydrochloride

Dichlorobenzyl alcohol - Sodium Benzoate

Natural extracts[1]

---

[1] icelandic lichen extract (Licheni islandicum extractum), marshmallow root extract (Althaeae radix extractum), sage extract (Salviae Folium Extractum), and Pelargonium sidoides.

D5. Which of the following formulations do you recommend for treating sore throat symptoms in children? Assign an order of preference to each (1 = most preferred formulation, 3 = least preferred formulation)

Spray

Hard candy

Mouthwash

Soft tab

D6. For which of the following throat and oral symptoms do your clients ask for advice? (indicate in percentage)

Ache

Itchy throat

Difficulty in swallowing

Dry throat

Mouth/throat burning

Redness of the throat

Tonsillitis

D7. Do you recommend Benzydamine Hydrochloride (Tantum Verde)?

Yes

No

D8. How often do you recommend Benzydamine Hydrochloride (Tantum Verde)? (Administered only to pharmacists who recommend Tantum Verde)

0 -| 20 %

20 -| 50 %

50 -| 70 %

> 70 %

D9. For each of the following diseases, indicate how often you recommend Benzydamine Hydrochloride (Tantum Verde) (indicate the percentage)

Gingivitis

Stomatitis

Conservative dental therapy

Extractive dental therapy

Sore throat

Other

D10. For which of the following symptoms reported by the customer do you recommend Benzydamine Hydrochloride (Tantum Verde)? (indicate in percentage)

Ache

Itchy and itchy throat

Difficulty in swallowing

Dry throat

Burning of the mouth

Redness of the throat

Tonsillitis

D11. Which formulation of Benzydamine Hydrochloride (Tantum Verde) do you recommend the most? (indicate the percentage)

Mouthwash 0.15%

Spray 0.15%

Spray 0.30%

D12. Do you usually provide information on the dosage and duration of treatment with Benzydamine Hydrochloride (Tantum Verde)?

Yes

No

D13 Which are the main characteristics that make you recommend Benzydamine Hydrochloride (Tantum Verde)?

Anesthetics

Analgesics

Anti-inflammatory

Antiseptics

D14. What is the percentage of consumers that ask you for advice / suggestions on Benzydamine Hydrochloride (Tantum Verde) and its use?

Yes

No

D15. In your experience, out of the TOTAL of customers who request / purchase Benzydamine Hydrochloride (Tantum Verde) in what percentage they choose it:

Self-management

On medical advice

On pediatrician advice

On pharmacist advice

Survey for General practitioners

D1. According to your knowledge, are the following constituents can be used for the topical treatment of sore throat symptoms and various inflammatory / irritative conditions of the oral cavity such as gingivitis, stomatitis? (For each answer, tick Yes or No)

Ketoprofen

Flurbiprofen

Ambroxol Chlorydrate

Dichlorobenzyl alcohol - Sodium Benzoate

Benzydamine Hydrochloride

Natural extracts[1]

D2. In general, which active ingredients do you prescribe / recommend for the topical treatment of sore throat symptoms and various inflammatory / irritative conditions of the oral cavity such as gingivitis, stomatitis? (For each answer, tick Yes or No)

Ketoprofen

Flurbiprofen

Ambroxol Chlorydrate

Dichlorobenzyl alcohol - Sodium Benzoate

Benzydamine Hydrochloride

Natural extracts[1]

D3. Based on your preference, order each of the following active ingredients for the topical treatment of sore throat symptoms and various inflammatory / irritative conditions of the oral cavity such as gingivitis, stomatitis and following extractive therapies

Ketoprofen

Flurbiprofen

Ambroxol Chlorydrate

Dichlorobenzyl alcohol - Sodium Benzoate

Benzydamine Hydrochloride

Natural extracts[1]

Others

D4. Your therapeutic approach involves (only one answer):

Start with topical medication

Start with topical medication and move on to systemic

No preference

D5. Which of the following formulations do you prescribe / recommend for the treatment of sore throat symptoms in adults? (Assign an order of preference to each)

Spray

Hard candy

Mouthwash

Soft tab

D6. Which of the following active ingredients do you prescribe / recommend for the topical treatment of sore throat symptoms in children? (Assign an order of preference to each)

Benzydamine Hydrochloride

Dichlorobenzyl alcohol - Sodium Benzoate

Natural extracts[1]

D7. Which of the following formulations do you prescribe / recommend for the treatment of sore throat symptoms in children? Assign an order of preference to each (1 = most preferred formulation, 3 = least preferred formulation)

Spray

Hard candy

Soft tab

D8. Do you prescribe / recommend Benzydamine hydrochloride (Tantum Verde)?

Yes

No

D9. How often do you prescribe / recommend Benzydamine hydrochloride (Tantum verde)? (only one answer) (Administered only to doctors who recommend/prescribe Tantum Verde)

0 -| 20 %

20 -| 50 %

50 -| 70 %

> 70 %

D10. For each of the following conditions, indicate the percentage of times you prescribe / recommend Benzydamine hydrochloride (Tantum Verde)

Gingivitis

Stomatitis

Conservative dental therapy

Extractive dental therapy

Sore throat

Other

D11. For each of the following patient-reported symptoms, indicate the percentage of times you prescribe / recommend Benzydamine hydrochloride (Tantum Verde)

Ache

Itchy and itchy throat

Difficulty in swallowing

Dry throat

Burning of the mouth

Redness of the throat

D12. For each of the following formulations of Benzydamine hydrochloride (Tantum Verde), indicate the percentage of times you prescribe / recommend it

Mouthwash 0.15%

Spray 0.15%

Spray 0.30%

P 3mg

D13. Do you usually provide information on the posology and duration of treatment with Benzydamine hydrochloride (Tantum Verde)?

Yes

No

D14. Which are the main characteristics that make you prescribe / recommend Benzydamine hydrochloride (Tantum Verde)? (indicate for all possible options the level of importance with a number from 1 to 5, e.g. 1: - Not very important - 5: Very important)

Anesthetics

Analgesics

Anti-inflammatory

Antiseptics

D15. Do you prescribe / recommend Benzydamine hydrochloride (Tantum Verde) in combination with other drugs?

Yes

No

D16. If yes, which are the other drugs that you prescribe/recommend in combination with Benzydamine hydrochloride (more than answer)

Anti-inflammatory

Antibiotics

Analgesics

Other

D17. Do you prescribe / recommend Benzydamine hydrochloride (Tantum Verde) for children?

Yes

No
