# Supplementary material for: Benzydamine hydrochloride for the treatment of sore throat and irritative/inflammatory conditions of the oropharynx: a cross-national survey among pharmacists and general practitioners
Source: BMC Prim Care. 2022 Jun 17;23:154. doi: 10.1186/s12875-022-01762-3 (PMC9205545; doi:10.1186/s12875-022-01762-3)
Supplement: Supplementary file 1 — Additional file 1. [file 12875_2022_1762_MOESM1_ESM.zip › Appendix 2 - Survey Data & Results.pdf]

Appendix 2

Questionnaire survey for Pharmacists and General Practitioners in 4 European countries (Italy, Germany, Russia and Poland)

Data & Results

1. Introduction

The objective of the study is, on one side, to verify the extent of the knowledge of Benzydamine hydrochloride (Tantum Verde) as a topical treatment for mouth conditions and, on the other side, to understand how it is used compared to other drugs.

To reach the research goals, 802 questionnaires were administered to pharmacists and general practitioners (in equal amounts) in four different countries (Russia, Poland, Italy and Germany) during the months of June, 2021.

This document reports the results of the aforementioned quantitative survey. The sampling plan provided for the administration of the questionnaire to 100 pharmacists and 100 GPs for each country. A total of 401 answers valid among GPs and 401 among Pharmacists were obtained. The questionnaires administered to the two categories of health workers differ slightly from each other, to take into account the different skills and professional activities. Copies of the two questionnaires, which were then translated into the respective languages of the countries involved, are shown in the Appendix (Italian version).

2. Summary of results

Both GP and pharmacists proved to have an excellent knowledge and mastery of the constituents effective against oral symptoms: more than 95% declare to know which are the effective treatments.

Among all the principles, Benzydamine hydrochloride is the most recognized as certainly suitable for the topical treatment of sore throat symptoms and various inflammatory / irritative conditions of the oral cavity such as gingivitis and stomatitis. It is recommended by about 90% of Pharmacists and prescribed by 80% of GPs. The latter indicate it as the preferred remedy in 21.3% of cases, a percentage almost equivalent to that of those who indicate Ketoprofen as the best treatment (21.9%), and higher than the percentage choosing Flurbiprofen as the most suitable one (19.3%). 308 out of 401 Pharmacists and 288 out of 401 GPs recommend Benzydamine, mainly to solve the ailments caused by sore throats and stomatitis, especially for its anti-inflammatory, analgesic and anesthetic characteristics.

Also in the pediatric field, Benzydamine hydrochloride is indicated: among GPs, a high percentage (about 40%) prescribes it like the remedies based on Dichlorobenzyl Alcohol-Sodium Benzoate, which are instead more often indicated by Pharmacists (44% against 37%).

3. Detailed findings

In the following sections, we provide detailed results for Pharmacists and GPs separately focusing on the most interesting results of the survey. Note that, with a slight abuse, the results are read in terms of percentages but in the tables there are relative frequencies (which add up to 1 and not to 100). We allow ourselves to do this thanks to the close relationship between these two quantities. For each analyzed question, we first report the

table and/or the corresponding figure followed by a short comment. All tables are reported in the Appendix.

3.1 PHARMACISTS

3.1.1 Drugs constituents: knowledge and recommendations

Table 3.1 – Based on your knowledge, are the following active ingredients usable for the topical medication of sore throat and inflammatory/irritative conditions of the mouth such as gingivitis or stomatitis?

Values	Ketoprofene	Flurbiprofene	Ambroxolo Cloridrato	Benzyl alcohol - sodium benzoate	Benzydamine hydrochloride	Natural extracts
Yes	0.763	0.818	0.895	0.918	0.953	0.731
No	0.202	0.157	0.087	0.072	0.037	0.185
Don't know	0.035	0.025	0.017	0.01	0.01	0.085

Figure 3.1 - Based on your knowledge, are the following active ingredients usable for the topical medication of sore throat and inflammatory/irritative conditions of the mouth such as gingivitis or stomatitis?

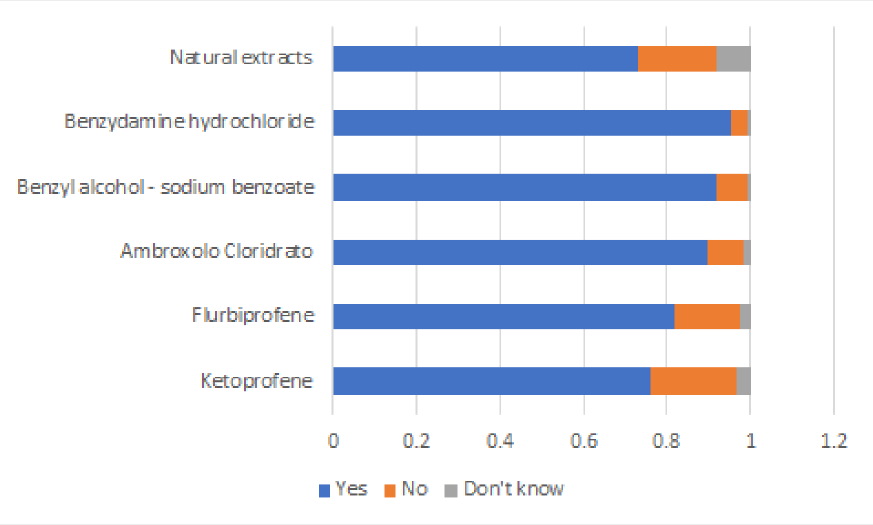

The interviewees show an excellent knowledge of the treatment for oral pain conditions in general and of Benzydamine hydrochloride, in particular. In fact, among the 6 possible constituents listed in the questionnaire, Benzydamine hydrochloride, is the most frequently recognized effective treatment for sore throat and pain conditions in the mouth, with more than 95% of the respondent pharmacists declaring it as usable in such situations. This is clearly shown in figure 3.1 which is derived from table 3.1.

Table 3.2 - In general, which active ingredients do you prescribed / recommended for the topical treatment of sore throat symptoms and various inflammatory / irritative conditions of the oral cavity such as gingivitis, stomatitis? (For each answer, tick Yes or No)

Values	Ketoprofene	Flurbiprofene	Ambroxolo Cloridrato	Benzyl alcohol - sodium benzoate	Benzydamine hydrochloride	Natural extracts
Yes	0.721	0.813	0.898	0.903	0.893	0.574
No	0.279	0.187	0.102	0.097	0.107	0.426

Figure 3.2 - In general, which active ingredients do you prescribed / recommended for the topical treatment of sore throat symptoms and various inflammatory / irritative conditions of the oral cavity such as gingivitis, stomatitis? (For each answer, tick Yes or No)

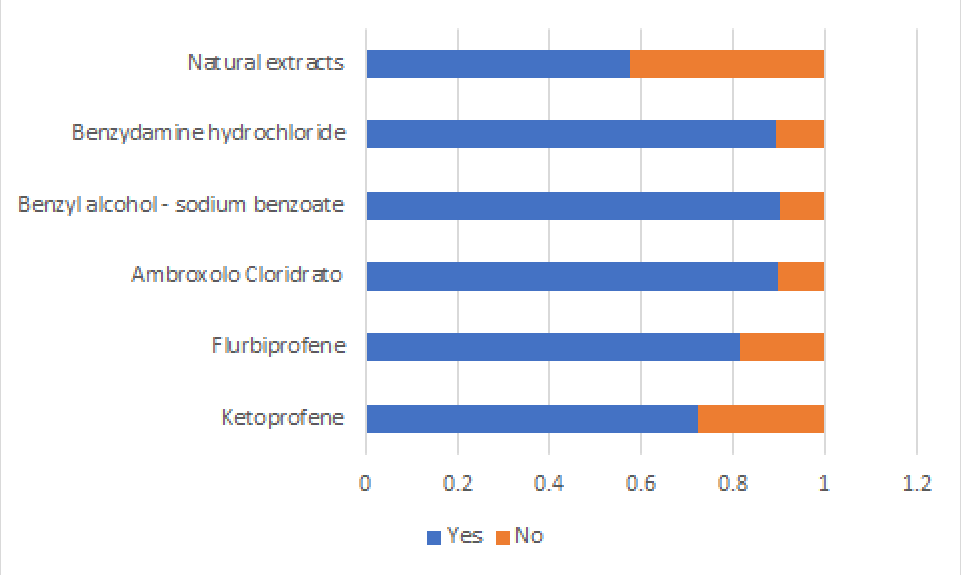

The three constituents most advised by the Pharmacists for the topical treatment of sore throat and other oral pathologies are Benzyl alcohol - sodium benzoate (more that 90% of the respondents advice it), Ambroxolo chloridrate (a little less than 90%), Benzydamine hydrochloride (89.3%). This proves a leading position for Benzydamine hydrochloride.

Table 3.3 - Do you recommend Benzydamine Hydrochloride (Tantum Verde)?

Values	n	n/N
Yes	308	0.768
No	93	0.232

Figure 3.3 - Do you recommend Benzydamine Hydrochloride (Tantum Verde)?

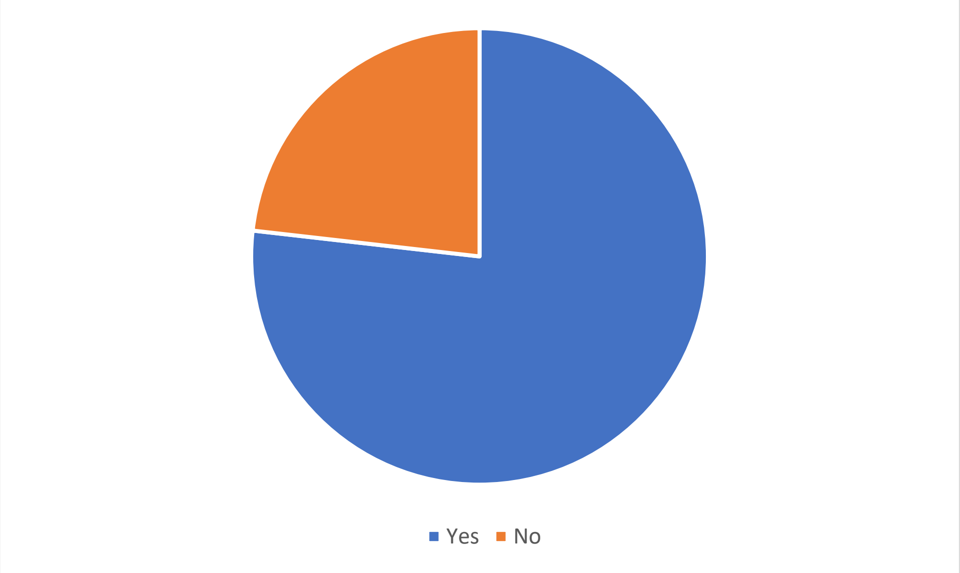

The great majority of pharmacists recommend Benzydamine Hydrochloride to their customers.

The following questions in this section have been administrated only to those answering “Yes” to the previous issue.

Table 3.4 - For each of the following pathological conditions, indicate how often you recommend Benzydamine Hydrochloride (Tantum Verde) (indicate the percentage)

	Gingivitis	Stomatitis	Conservative dental therapy	Extractive dental therapy	Sore throat	Other
Min.	0	0	0	0	0	0
1st Quart.	10	10	10	10	15	0
Median	20	16	10	10	30	0
Mean	20.28	16.23	13.79	12.78	32.89	4.04
3rd Quart.	25	20	20	20	50	5
Max	100	50	50	90	100	60

Figure 3.4 – For each of the following pathological conditions, indicate how often you recommend Benzydamine Hydrochloride (Tantum Verde) (indicate the percentage)

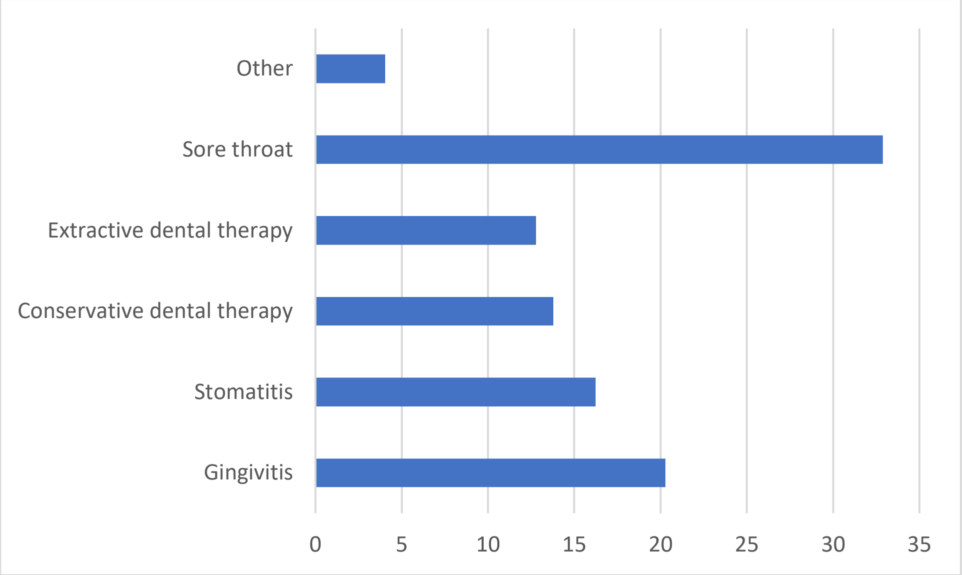

Sore throat is by far the pathological condition for which pharmacists more often recommend the use of Benzydamine Hydrochloride.

Figure 3.5 - For which of the following symptoms reported by the customer do you recommend Benzydamine Hydrochloride (Tantum Verde)? (indicate in percentage)

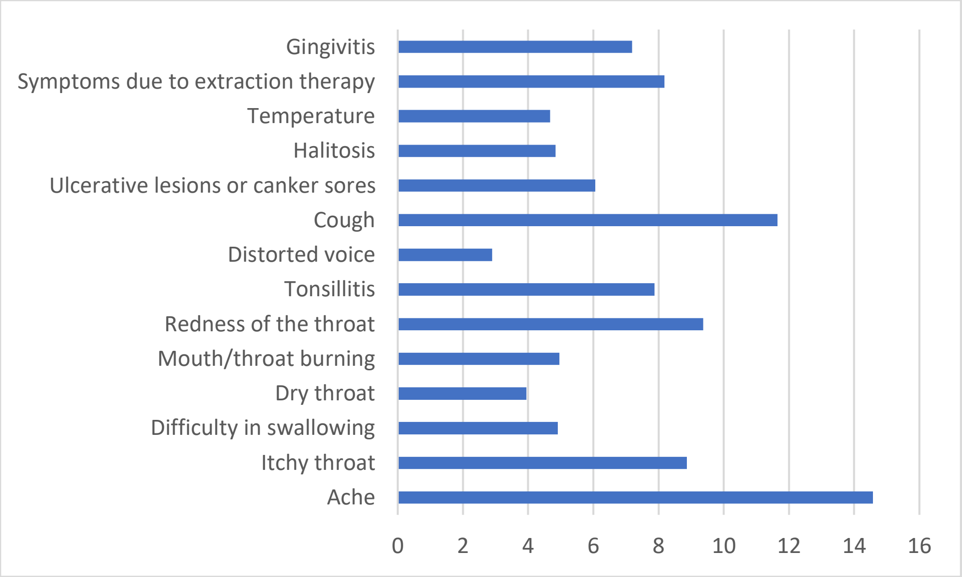

Ache and cough are the symptoms for which more often the pharmacists suggest the use of Benzydamine Hydrochloride.

Table 3.5 - Which are the main characteristics that make you recommend Benzydamine Hydrochloride (Tantum Verde)? (1=Not very important, 5=Very important)

Values	Anesthetics	Analgesics	Anti-inflammatory	Antiseptics
1 Not very important	0.036	0.068	0.049	0.049
2	0.117	0.120	0.075	0.058
3	0.201	0.182	0.195	0.153
4	0.289	0.282	0.263	0.416
5 Very important	0.357	0.347	0.419	0.325

Figure 3.6 - Which are the main characteristics that make you recommend Benzydamine Hydrochloride (Tantum Verde)? (frequency of 5 Very important)

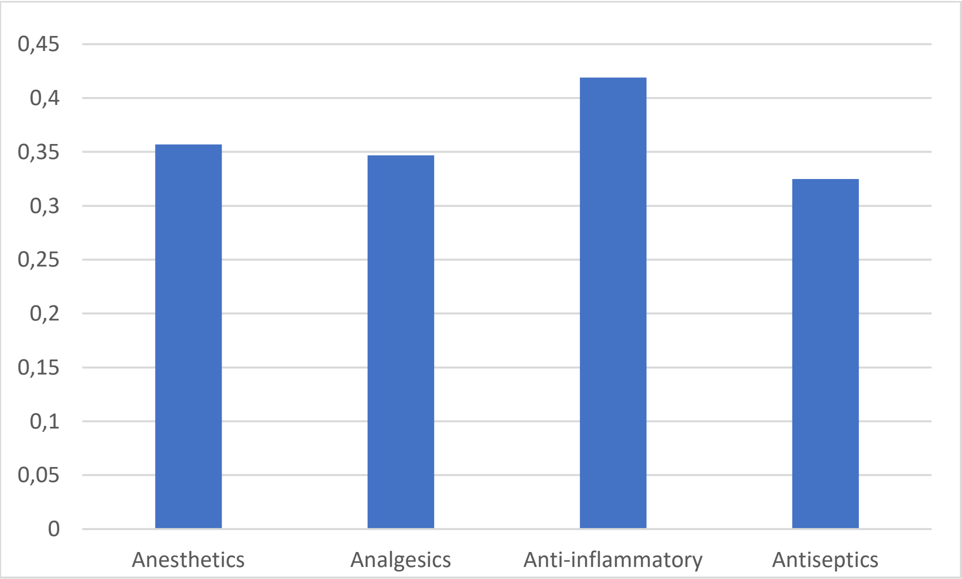

The anti-inflammatory property is the most important one in the choice of the active ingredient of interest, followed by the pain-relieving ones.

3.1.2 Oral treatment usage

Table 3.6 - Which of the following formulations do you prescribe / recommend for the treatment of sore throat symptoms in adults? Assign an order of preference to each (1 = most preferred, 4 = least preferred)

Preference	Spray	Hard candy	Mouthwash	Soft tab
1 Highest	0.534	0.292	0.117	0.057
2	0.182	0.272	0.362	0.185
3	0.219	0.307	0.272	0.202
4 Lowest	0.065	0.130	0.249	0.556
Avg score[1]	1.815	2.274	2.653	3.257

Pharmacists' most recommended way of administering sore throat treatments is through medications in spray form (53.4% of the respondents point it as the favorite mode), followed by hard candy (29.2%).

3.1.3 Treatment in children

Table 3.7 - Which of the following active ingredients do you recommend for the topical treatment of sore throat symptoms in children? Assign an order of preference to each (1 = most preferred, 3 = least preferred)

Preference	Benzydamine	Diclorobenzil	Natural extracts
1 Highest	0.369	0.444	0.187
2	0.392	0.481	0.127
3 Lowest	0.239	0.075	0.686
Avg score	1.870	1.631	2.499

For sore throat treatment in the pediatric population, the preferred constituent of pharmacists is Diclorobenzil, with 44.4% of the respondents pointing it as the highest preferred one and an overall average score of 1.631. Benzydamine closely follows with an average score of 1.870, making it the second preferred active ingredient.

---

[1] The score ranges from 1 (best score) to 4 (worst score). Thus, the lower the average score is, the best the position of the item in the overall ranking. The same applies to the following tables.

Table 3.8 - Which of the following formulations do you recommend for treating sore throat symptoms in children? Assign an order of preference to each (1 = most preferred, 3 = least preferred)

Preference	Spray	Hard candy	Soft tab
1 Highest	0.352	0.317	0.332
2	0.274	0.414	0.312
3 Lowest	0.374	0.269	0.357
Avg score	2.022	1.953	2.025

As for the preferred formulation, spray is the one most indicated by the Pharmacists also for treating children (35.2%), closely followed by soft tabs.

3.1.4 Consumer behaviour

Table 3.9 - In your experience, out of the TOTAL of customers who request / purchase Benzydamine Hydrochloride (Tantum Verde) in what percentage they choose it:

	Self-management	On doctor advice	On pediatrician advice	On pharmacist advice
Min.	0	0	0	0
1st Quart.	25	20	10	10
Median	35	20	20	10
Mean	40.03	24.24	18.98	16.75
3rd Quart.	55	30	25	20
Max	100	60	70	100

Figure 3.7- In your experience, out of the TOTAL of customers who request / purchase Benzydamine Hydrochloride (Tantum Verde) in what percentage they choose it:

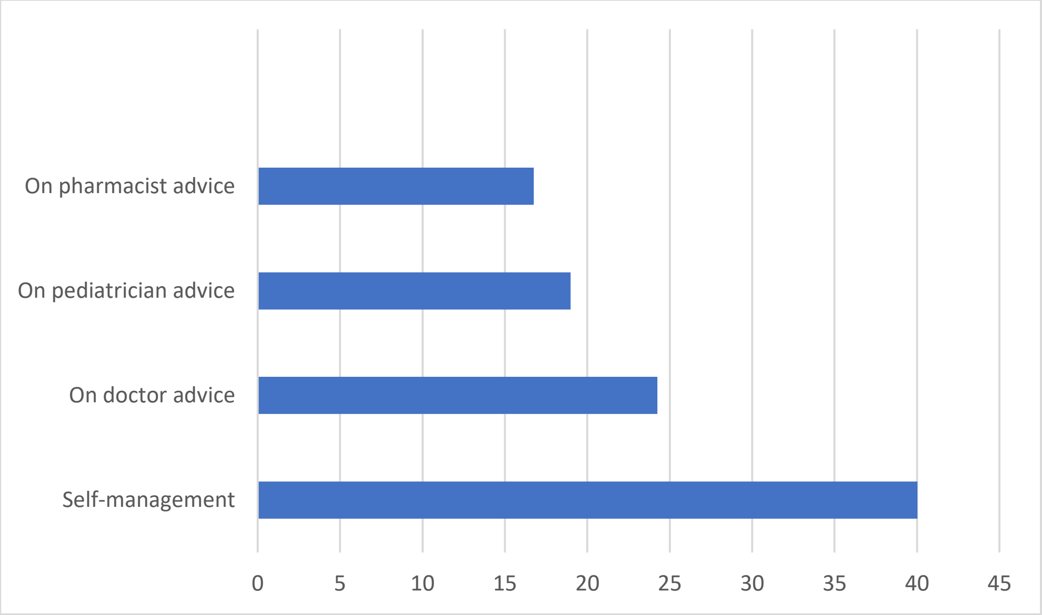

End users appear very familiar with Benzydamine Hydrochloride, as evidenced by the fact that on average 40% of people require it as a self-medication.

3.2 GENERAL PRACTICTIONERS

3.2.1 Drugs constituents: knowledge and recommendations

Table 3.10 - Based on your knowledge, are the following active ingredients usable for the topical medication of sore throat and inflammatory/irritative conditions of the mouth such as gingivitis or stomatitis?

Values	Ketoprofene	Flurbiprofene	Ambroxolo Cloridrato	Benzyl alcohol - sodium benzoate	Benzydamine hydrochloride	Natural extracts
Yes	0.751	0.803	0.855	0.853	0.923	0.626
No	0.202	0.177	0.130	0.127	0.065	0.269
Don't know	0.047	0.020	0.015	0.020	0.012	0.105

Figure 3.8 - Based on your knowledge, are the following active ingredients usable for the topical medication of sore throat and inflammatory/irritative conditions of the mouth such as gingivitis or stomatitis?

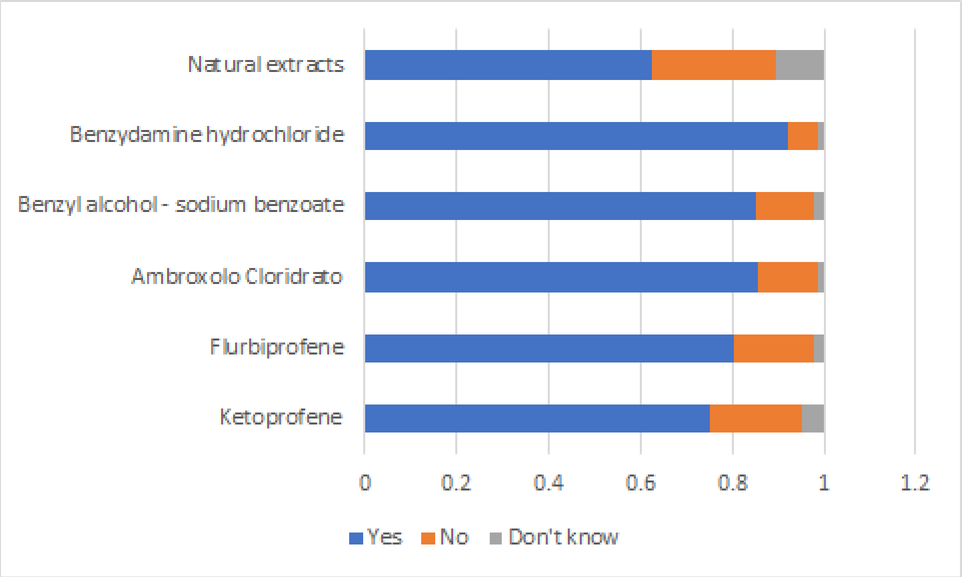

Almost all the interviewed doctors (92.3%) indicated Benzydamine hydrochloride as an adequate treatment for sore throat. It is by far the highest percentage followed by that of those inidicate Ambroxolo Cloridrate with almost ten points of difference.

Table 3.11 – In general, which active ingredients do you prescribed / recommended for the topical treatment of sore throat symptoms and various inflammatory / irritative conditions of the oral cavity such as gingivitis, stomatitis? (For each answer, tick Yes or No)

Values	Ketoprofene	Flurbiprofene	Ambroxolo Cloridrato	Benzyl alcohol - sodium benzoate	Benzydamine hydrochloride	Natural extracts
Yes	0.698	0.761	0.813	0.835	0.808	0.491
No	0.302	0.239	0.187	0.165	0.192	0.509

Figure 3.9 - In general, which active ingredients do you prescribed / recommended for the topical treatment of sore throat symptoms and various inflammatory / irritative conditions of the oral cavity such as gingivitis, stomatitis? (For each answer, tick Yes or No)

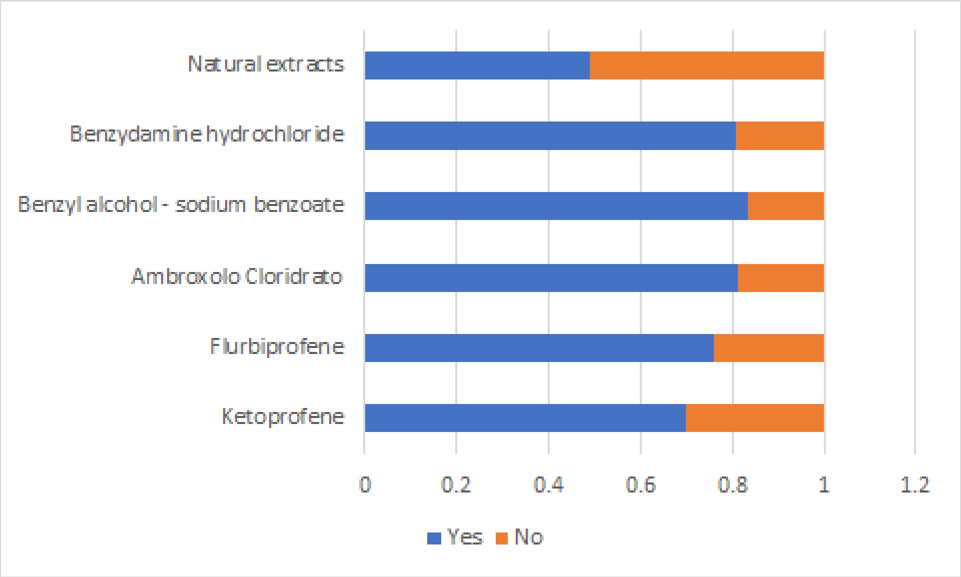

Benzydamine hydrochloride is recommended by a very high percentage of doctors (80.8%) in the topical treatment of oral cavity (see table 3.11). It is surpassed only by Benzyl alcohol - sodium benzoate which is prescribed by a slightly higher percentage of doctors.

Table 3.12 - Based on your preference, order each of the following active ingredients for the topical treatment of sore throat symptoms and various inflammatory / irritative conditions of the oral cavity such as gingivitis, stomatitis and following extractive therapies (1 = most preferred, 7 = least preferred)

Value	Ketoprofene	Flurbiprofene	Ambroxolo Cloridrato	Benzyl alcohol	Benzydamine hydrochloride	Natural extracts	Others
1 Most pref.	0.219	0.193	0.114	0.127	0.213	0.126	0.004
2	0.232	0.168	0.212	0.225	0.099	0.044	0.027
3	0.157	0.236	0.28	0.149	0.132	0.028	0.022
4	0.157	0.204	0.172	0.256	0.165	0.036	0.013
5	0.195	0.161	0.154	0.154	0.289	0.041	0.009
6	0.040	0.035	0.053	0.076	0.094	0.692	0.031
7 Least pref	0.000	0.003	0.015	0.013	0.008	0.033	0.894
Avg score	2.995	3.085	3.260	3.365	3.530	5.031	6.664

As shown in table 3 although Benzydamine hydrochloride is recognized as one of the preferred ingredients to be effective in the treatment of sore throat (21.3% of GPs indicate it at the highest position, a percentage very close to that of Ketaprofene), the average score assigned to it is worse than that assigned to the other ingredients except for natural or other remedies (i.e.: 3.530 against 2.995 of Ketaprofene or 3.085 for Flurbiprofene). This means that Benzydamine hydrochloride is positioned overall worse than the other ingredients in the doctors' preference list.

Table 3.13 - Do you prescribe / recommend Benzydamine hydrochloride (Tantum Verde)?

Values	n	n/N
Yes	288	0.718
No	113	0.282

Figure 3.10 - Do you prescribe / recommend Benzydamine hydrochloride (Tantum Verde)?

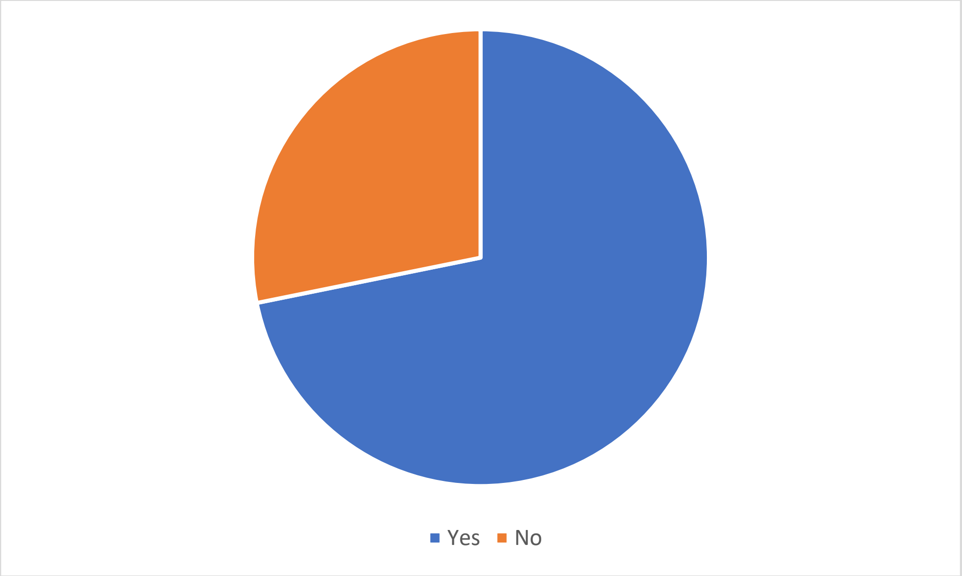

More than 70% of the interviewed GPs usually prescribe the active ingredient of interest.

The following questions in this section have been administrated only to those answering "Yes" to the previous issue.

Table 3.14 - For each of the following pathological conditions, indicate how often you recommend Benzydamine Hydrochloride (Tantum Verde) (indicate the percentage)

	Gingivitis	Stomatitis	Conservative dental therapy	Extractive dental therapy	Sore throat	Other
Min.	0	0	0	0	0	0
1st Quart.	10	10	10	10	10	0
Median	20	20	15	20	20	0
Mean	19.25	16.73	16.23	15.84	27.52	4.43
3rd Quart.	25	20	20	20	40	5
Max	100	50	100	100	100	60

Figure 3.11 - For each of the following pathological conditions, indicate how often you recommend Benzydamine Hydrochloride (Tantum Verde) (indicate the percentage)

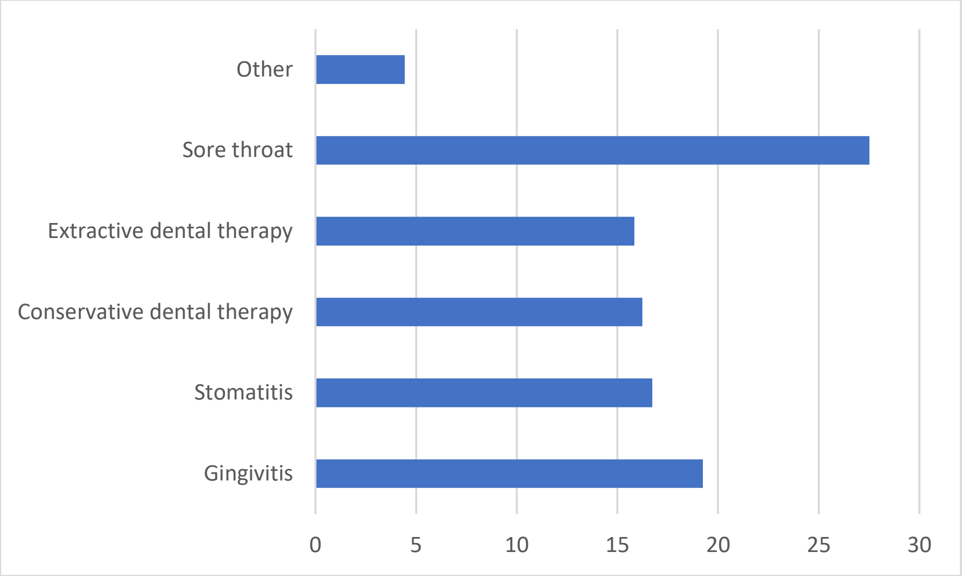

As for the Pharmacists, sore throat is by far the pathological condition for which Doctors more often recommend the use of Benzydamine Hydrochloride.

Figure 3.12 - For which of the following symptoms reported by the patient do you recommend Benzydamine Hydrochloride (Tantum Verde)? (indicate in percentage)

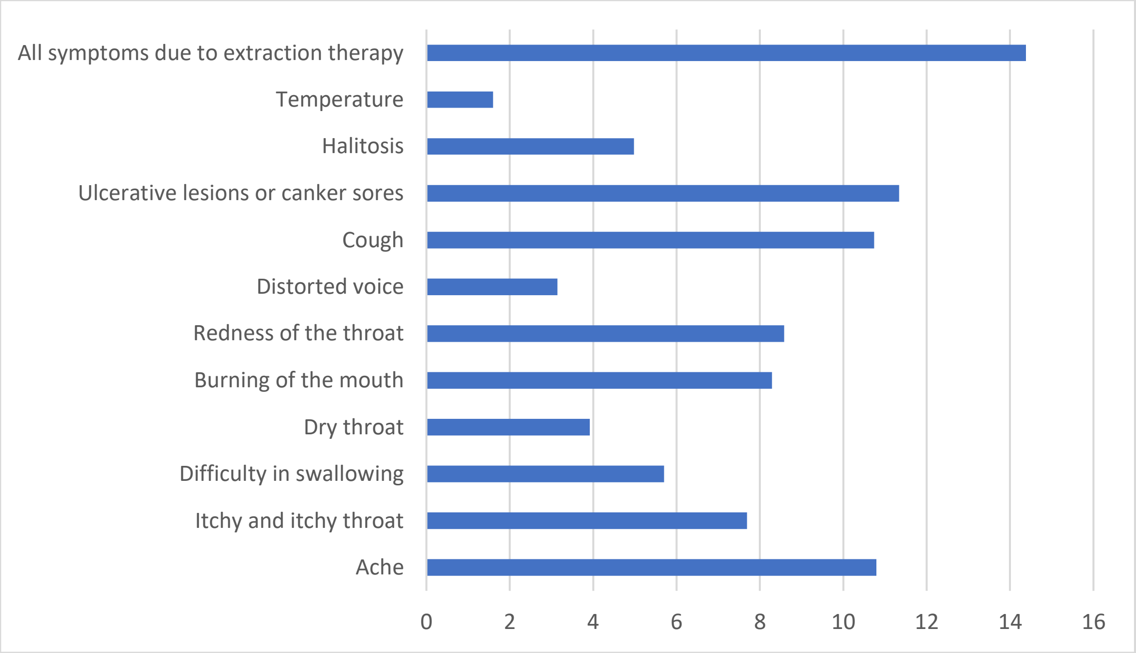

Differently from the Pharmacists, the symptom for which Doctors most often give indications to use Tantum Verde are those related to extraction therapy.

Table 3.15 - Which are the main characteristics that make you recommend Benzydamine Hydrochloride (Tantum Verde)? (1=Not very important, 5=Very important)

Values	Anesthetics	Analgesics	Anti-inflammatory	Antiseptics
1 Not very important	0.125	0.076	0.052	0.049
2	0.132	0.142	0.132	0.066
3	0.142	0.226	0.229	0.170
4	0.274	0.274	0.222	0.410
5 Very important	0.326	0.281	0.365	0.306

Figure 3.13 - Which are the main characteristics that make you recommend Benzydamine Hydrochloride (Tantum Verde)? (frequency of 5 Very important)

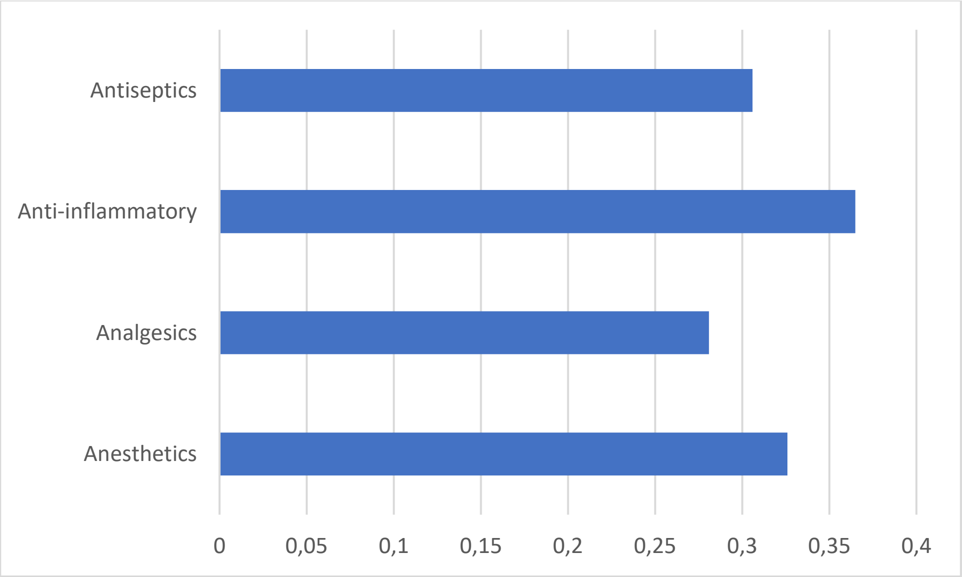

Again, the most appreciated characteristics of the ingredient Benzydamine Hydrochloride is its ant-inflammatory features, followed by the anesthetic ones. More than the Pharmacists, the GPs recognize the antiseptics properties.

3.2.2 Oral treatment usage

Table 3.16 - Which of the following formulations do you prescribe / recommend for the treatment of sore throat symptoms in adults? Assign an order of preference to each (1 = most preferred, 4 = least preferred)

Preference	Spray	Hard candy	Mouthwash	Soft tab
1 Highest	0.566	0.287	0.105	0.042
2	0.125	0.274	0.414	0.187
3	0.242	0.379	0.224	0.155
4 Lowest	0.067	0.060	0.257	0.616
Avg score	1.810	2.212	2.633	3.344

As for the Pharmacists, also among the GPs the preferred formulation for the administration of topical treatment is the spray.

3.2.3 Treatment in children

Table 3.17 - Which of the following active ingredients do you prescribe / recommend for the topical treatment of sore throat symptoms in children? Assign an order of preference to each (1 = most preferred, 4 = least preferred)

Value	Benzydamine hydrochloride	Diclorobenzil Alcool 2 mg, Sodio Benzoato 20 mg	Natural extracts
1 Most pref	0.397	0.406	0.195
2	0.401	0.441	0.155
3	0.202	0.15	0.643
4 Least pref	0.000	0.002	0.007
Avg score	1.805	1.748	2.464

A very similar percentage of GPs indicate Diclorobenzil Alcool/Sodio Benzoato 20 mg and Benzydamine hydrochloride as the first preference (40.6% and 39.7%, respectively) compared to natural extracts. However, the average score is a bit worse.

Table 3.18 - Which of the following formulations do you prescribe / recommend for the treatment of sore throat symptoms in children? Assign an order of preference to each (1 = most preferred, 3 = least preferred)

Preference	Spray	Hard candy	Soft tab
1 Highest	0.384	0.266	0.349
2	0.377	0.407	0.216
3 Lowest	0.239	0.327	0.435
Avg score	1.854	2.060	2.085

Again, the preferred formulation is the spray treatment mode.

4. Analysis by country

4.1 Pharmacists

In this section we compare the responses between the samples of the different countries involved in the survey. The analytical tables are flanked by graphs to highlight the responses for which the greatest intercountry differences are observed.

Table 4.1 - Based on your knowledge, are the following active ingredients usable for the topical medication of sore throat and inflammatory/irritative conditions of the mouth such as gingivitis or stomatitis? (Frequency distribution)

Value	Country	Ketoprofene	Flurbiprofene	Ambroxolo Cloridrato	Alcool Benzilico - sodio benzoato	Benzidamina Cloridrato	Natural extracts
Yes	DE	0.62	0.66	0.82	0.88	0.89	0.55
No	DE	0.35	0.31	0.18	0.11	0.09	0.29
Don't know	DE	0.03	0.03	0.00	0.01	0.02	0.16
Yes	ITA	0.99	0.98	0.96	0.97	1.00	0.86
No	ITA	0.01	0.02	0.03	0.03	0.00	0.11
Don't know	ITA	0.00	0.00	0.01	0.00	0.00	0.03
Yes	PL	0.75	0.86	0.96	0.94	0.94	0.73
No	PL	0.21	0.11	0.03	0.05	0.04	0.17
Don't know	PL	0.04	0.03	0.01	0.01	0.02	0.10
Yes	RUS	0.69	0.77	0.84	0.88	0.98	0.78
No	RUS	0.24	0.19	0.11	0.10	0.02	0.17
Don't know	RUS	0.07	0.04	0.05	0.02	0.00	0.05

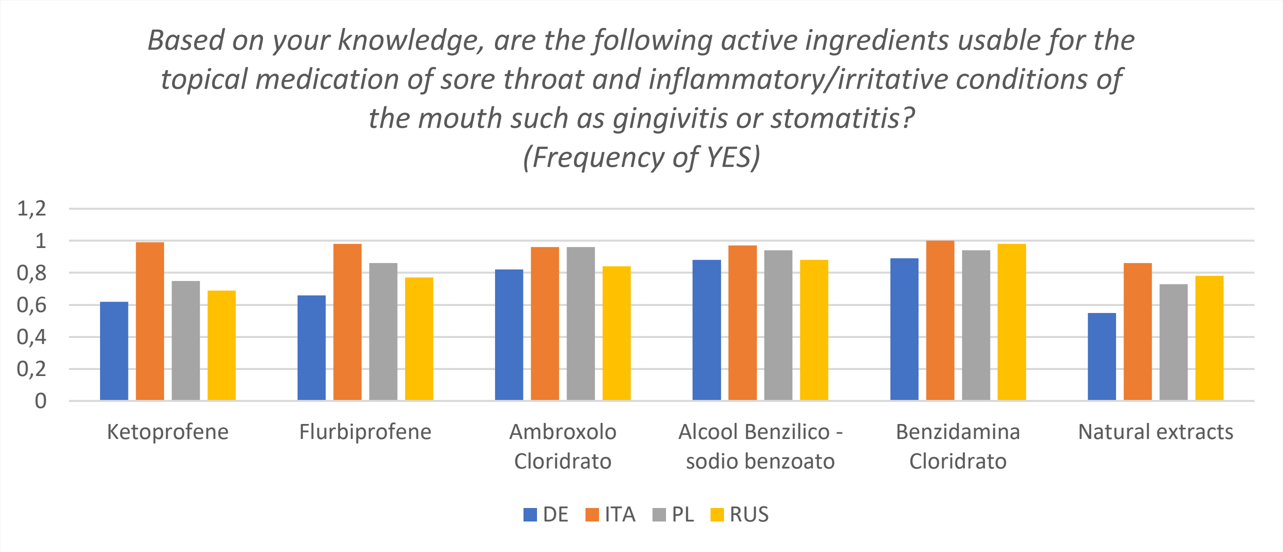

Table 4.2 - In general, which active ingredients prescribed / recommended for the topical treatment of sore throat symptoms and various inflammatory / irritative conditions of the oral cavity such as gingivitis, stomatitis? (For each answer, tick Yes or No)

Values	Country	Ketoprofene	Flurbiprofene	Ambroxolo Cloridrato	Benzyl alcohol - sodium benzoate	Benzydamine hydrochloride	Natural extracts
Yes	DE	0.54	0.64	0.80	0.83	0.81	0.46
No	DE	0.46	0.36	0.20	0.17	0.19	0.54
Yes	ITA	0.95	0.98	0.94	0.96	0.98	0.70
No	ITA	0.04	0.01	0.05	0.03	0.01	0.29
Yes	PL	0.70	0.87	0.93	0.93	0.86	0.49
No	PL	0.31	0.14	0.08	0.08	0.15	0.52
Yes	RUS	0.68	0.75	0.91	0.88	0.91	0.63
No	RUS	0.31	0.24	0.08	0.11	0.08	0.36

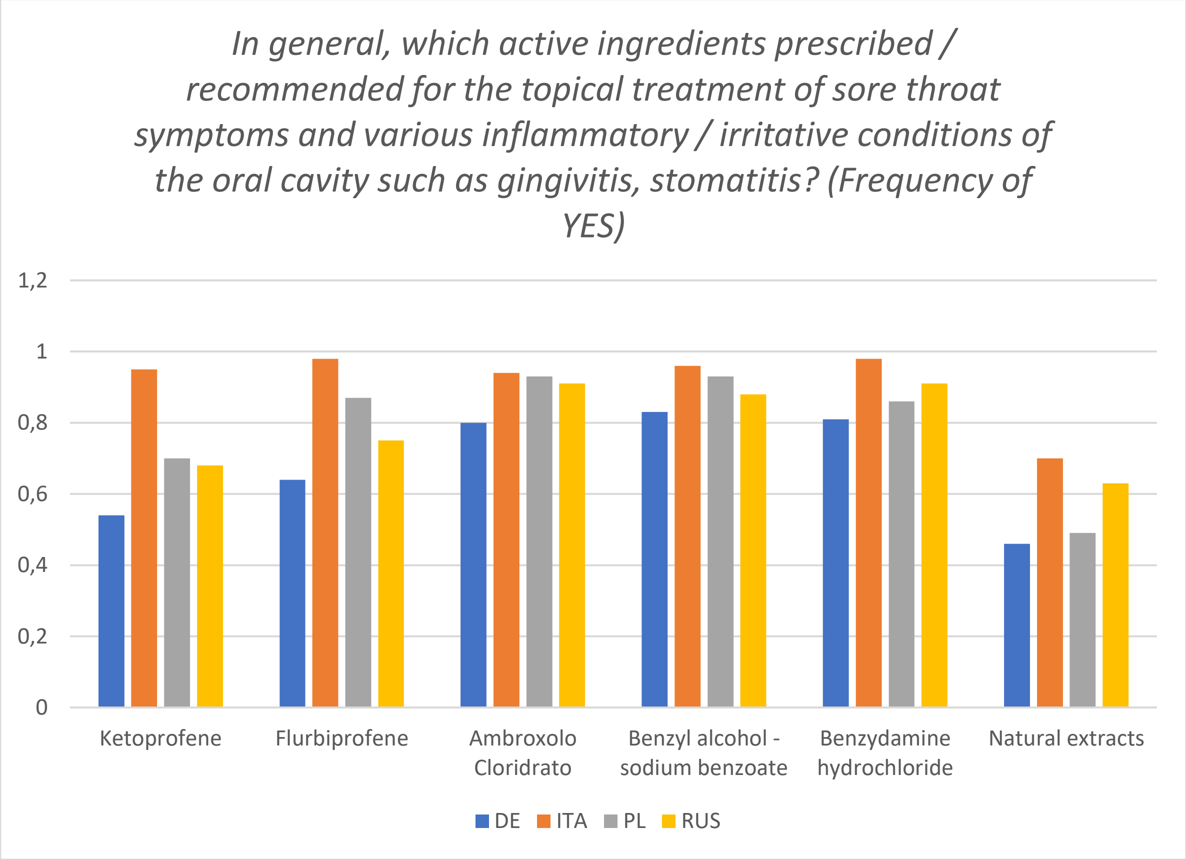

Table 4.3 - Which of the following formulations do you recommend for the treatment of sore throat symptoms in adults? Assign an order of preference to each (1 = most preferred, 4 = least preferred)

Preference	Country	Spray	Hard candy	Mouthwash	Soft tablet
1 Highest	DE	0.60	0.18	0.15	0.07
2	DE	0.15	0.36	0.27	0.22
3	DE	0.11	0.37	0.36	0.16
4 Lowest	DE	0.14	0.09	0.22	0.55
Average score	DE	1.79	2.37	2.65	3.19
1 Highest	ITA	0.48	0.38	0.11	0.03
2	ITA	0.11	0.15	0.57	0.17
3	ITA	0.38	0.36	0.13	0.13
4 Lowest	ITA	0.03	0.11	0.19	0.67
Average score	ITA	1.96	2.20	2.40	3.44
1 Highest	PL	0.58	0.26	0.09	0.08
2	PL	0.19	0.37	0.30	0.15
3	PL	0.19	0.24	0.27	0.31
4 Lowest	PL	0.05	0.14	0.35	0.47
Average score	PL	1.71	2.26	2.87	3.16
1 Highest	RUS	0.48	0.35	0.12	0.05
2	RUS	0.28	0.21	0.31	0.20
3	RUS	0.20	0.26	0.33	0.21
4 Lowest	RUS	0.04	0.18	0.24	0.54
Average score	RUS	1.80	2.27	2.69	3.24

Table 4.4 - Which of the following active ingredients do you recommend for the topical treatment of sore throat symptoms in children? Assign an order of preference to each (1 = most preferred, 4 = least preferred)

Preference	Country	Benzidamina	Diclorobenzil	Natural extracts
1 Highest	DE	0.39	0.43	0.18
2	DE	0.41	0.48	0.11
3 Lowest	DE	0.20	0.09	0.70
Average score	DE	1.81	1.66	2.53
1 Highest	ITA	0.42	0.46	0.12
2	ITA	0.36	0.51	0.13
3 Lowest	ITA	0.22	0.03	0.75
Average score	ITA	1.80	1.57	2.63
1 Highest	PL	0.33	0.52	0.16
2	PL	0.51	0.43	0.07
3 Lowest	PL	0.17	0.06	0.78
Average score	PL	1.84	1.54	2.61
1 Highest	RUS	0.34	0.37	0.29
2	RUS	0.29	0.51	0.20
3 Lowest	RUS	0.37	0.12	0.51
Average score	RUS	2.03	1.75	2.22

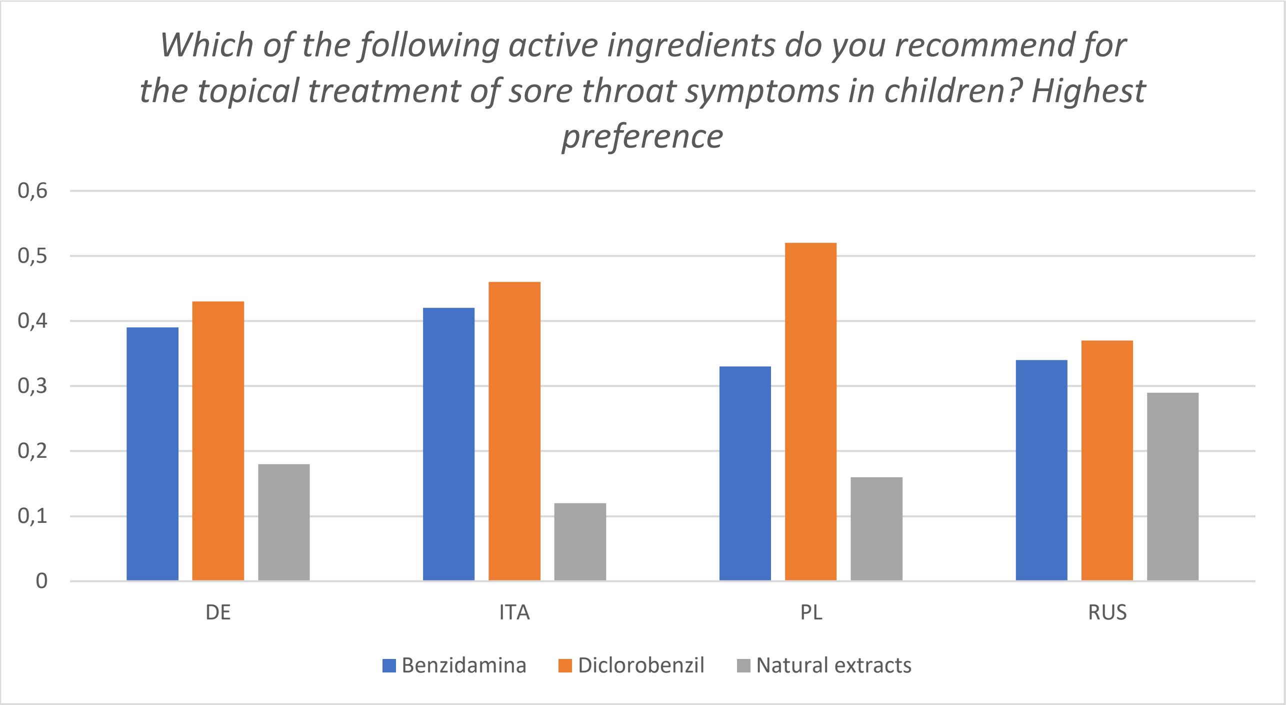

Table 4.5 - Which of the following formulations do you recommend for treating sore throat symptoms in children? Assign an order of preference to each (1 = most preferred, 3 = least preferred)

Preference	Country	Spray	Hard candy	Soft tabs
1 Highest	DE	0.54	0.29	0.17
2	DE	0.28	0.46	0.26
3 Lowest	DE	0.18	0.25	0.56
Average score	DE	1.64	1.96	2.40
1 Highest	ITA	0.24	0.34	0.42
2	ITA	0.24	0.34	0.42
3 Lowest	ITA	0.52	0.32	0.16
Average score	ITA	2.28	1.98	1.74
1 Highest	PL	0.30	0.32	0.39
2	PL	0.34	0.43	0.24
3 Lowest	PL	0.37	0.26	0.38
Average score	PL	2.07	1.94	1.99
1 Highest	RUS	0.33	0.32	0.35
2	RUS	0.24	0.43	0.33
3 Lowest	RUS	0.43	0.25	0.32
Average score	RUS	2.10	1.93	1.97

Table 4.6 - For which of the following throat and oral symptoms do your clients ask for advice? (indicate in percentage)

Country: Germany

	Ache	Itchy throat	Difficulty in swallowing	Dry throat	Mouth throat burning	Redness of the throat	Tonsillitis
Min.	0	0	0	0	0	0	0
1st Qu.	0	0	0	0	0	1.5	5
Median	10	5	5	0	5	5	10
Mean	8.67	5.49	5.69	4.06	7.26	7.51	10.19
3rd Qu.	10	10	10	5	10	10	10
Max.	30	30	30	30	20	30	30

	Distorted voice	Cough	Ulcerative lesions or canker sores	Halitosis	Temperature	Symptoms due to extraction therapy	Unknown
Min.	0	0	0	0	0	0	0
1st Qu.	0	0	5	0	0	0	0.75
Median	0	9	10	5	0	5	10
Mean	3.08	7.78	10.02	6.74	4.14	6.86	12.51
3rd Qu.	5	10	10	10	6.25	10	20
Max.	30	30	50	40	30	50	100

Country: Russia

	Ache	Itchy throat	Difficulty in swallowing	Dry throat	Mouth throat burning	Redness of the throat	Tonsillitis
Min.	0	0	0	0	0	0	0
1st Qu.	10	5	0	0	0	3	4.75
Median	10	10	5	4	5	5	6
Mean	15.41	10.37	5.79	3.8	4.51	8.39	7.24
3rd Qu.	24	16	10	5	7	10	10
Max.	40	30	50	30	30	30	35

	Distorted voice	Cough	Ulcerative lesions or canker sores	Halitosis	Temperature	Symptoms due to extraction therapy	Unknown
Min.	0	0	0	0	0	0	0
1st Qu.	0	5	2	0	0	4.75	0
Median	2.5	8	5	4.5	1	5	5
Mean	3.76	11.64	6.86	6.1	3.27	7.33	5.53
3rd Qu.	5	20	10	9	5	10	10
Max.	30	50	35	70	30	30	30

Country: Poland

	Ache	Itchy throat	Difficulty in swallowing	Dry throat	Mouth throat burning	Redness of the throat	Tonsillitis
Min.	0	0	0	0	0	0	0
1st Qu.	5	2	0	0	0	5	5
Median	10	5	5	5	5	5	10
Mean	12.03	5.05	4.90	4.89	6.19	8.43	8.74
3rd Qu.	20	5	5	5	10	10	10
Max.	100	20	30	30	100	30	50

	Distorted voice	Cough	Ulcerative lesions or canker sores	Halitosis	Temperature	Symptoms due to extraction therapy	Unknown
Min.	0	0	0	0	0	0	0
1st Qu.	0	5	5	0	0	5	5
Median	5	10	5	5	5	10	10
Mean	3.45	11.90	6.57	4.26	6.22	10.05	7.33
3rd Qu.	5	15	10	5	10	10	10
Max.	20	50	20	20	20	90	20

Country: Italy

	Ache	Itchy throat	Difficulty in swallowing	Dry throat	Mouth throat burning	Redness of the throat	Tonsillitis
Min.	0	0	0	0	0	0	0
1st Qu.	10	5	0	0	0	6	0
Median	20	10	2.5	1	0	10	5
Mean	22.2	14.61	3.24	3.05	1.88	13.16	5.32
3rd Qu.	30	25	5	5	3	20	10
Max.	50	70	20	30	20	40	20

	Distorted voice	Cough	Ulcerative lesions or canker sores	Halitosis	Temperature	Symptoms due to extraction therapy	Unknown
Min.	0	0	0	0	0	0	0
1st Qu.	0	8.75	0	0	0	5	0
Median	0	15	0	0	2	10	2
Mean	1.32	15.27	0.8	2.27	5.05	8.45	3.38
3rd Qu.	2	20	2	5	5	11	5
Max.	5	50	10	25	70	40	20

Table 4.7 - Do you recommend Benzydamine Hydrochloride (Tantum Verde)?

Values	Country	n	n/N
Yes	DE	82	0.82
No	DE	18	0.18
Yes	ITA	70	0.69
No	ITA	30	0.30
Yes	PL	78	0.78
No	PL	23	0.23
Yes	RUS	78	0.77
No	RUS	22	0.22

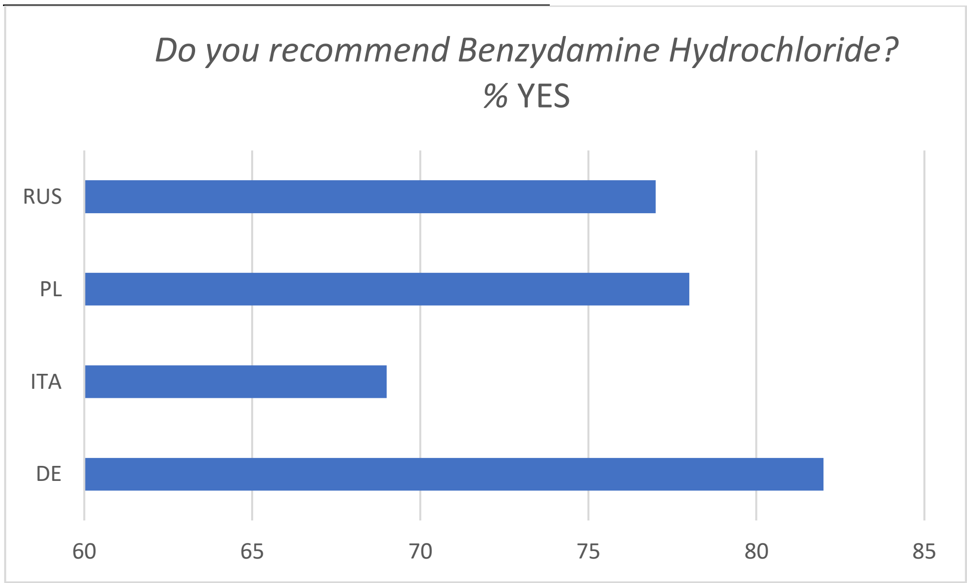

(Administered only to pharmacists who recommend Tantum Verde)

Table 4.8 -. How often do you recommend Benzydamine Hydrochloride (Tantum Verde)?

Values	Country	n	n/N
0 -| 20 %	DE	10	0.122
20 -| 50 %	DE	29	0.414
50 -| 70 %	DE	30	0.385
> 70 %	DE	13	0.167
0 -| 20 %	ITA	9	0.110
20 -| 50 %	ITA	19	0.271
50 -| 70 %	ITA	37	0.474
> 70 %	ITA	5	0.064
0 -| 20 %	PL	18	0.220
20 -| 50 %	PL	24	0.343
50 -| 70 %	PL	28	0.359
> 70 %	PL	8	0.103
0 -| 20 %	RUS	13	0.159
20 -| 50 %	RUS	26	0.371
50 -| 70 %	RUS	35	0.449
> 70 %	RUS	4	0.051

Table 4.9 - For each of the following pathological conditions, indicate how often you recommend Benzydamine Hydrochloride (Tantum Verde) (indicate the percentage)

Country: Germany	Gingivitis	Stomatitis	Conservative dental therapy	Extractive dental therapy	Sore throat	Other
Min.	0	0	0	0	0	0
1st Qu.	10	10	10	0	10	0
Median	20	20	10	10	30	0
Mean	22.012	18.537	13.293	10.671	33.049	2.439
3rd Qu.	22	20	20	10	50	0
Max.	100	50	50	90	100	60
NA's	18	18	18	18	18	18

Country: Italy	Gingivitis	Stomatitis	Conservative dental therapy	Extractive dental therapy	Sore throat	Other
Min.	2	0	0	2	0	0
1st Qu.	10	5	3.5	5	21.25	0
Median	20	10	10	10	40	3.5
Mean	20.457	10.614	10.814	10.786	42.714	4.614
3rd Qu.	25	15	15	15	60	5.75
Max.	70	25	50	30	90	15
NA's	30	30	30	30	30	30

Country: Poland	Gingivitis	Stomatitis	Conservative dental therapy	Extractive dental therapy	Sore throat	Other
Min.	0	0	0	0	0	0
1st Qu.	10	10	10	10	20	0
Median	20	20	10	10	30	0
Mean	20.064	18.205	14.359	15.513	30.513	1.346
3rd Qu.	25	20	20	20	40	0
Max.	50	50	50	50	80	20
NA's	23	23	23	23	23	23

Country: Russia	Gingivitis	Stomatitis	Conservative dental therapy	Extractive dental therapy	Sore throat	Other
Min.	0	0	0	0	0	0
1st Qu.	11.25	10	10	10	15	0
Median	20	18	15	10	20	5
Mean	18.526	16.859	16.397	14.051	26.282	7.885
3rd Qu.	22	20	20	20	40	10
Max.	50	35	30	50	70	40
NA's	22	22	22	22	22	22

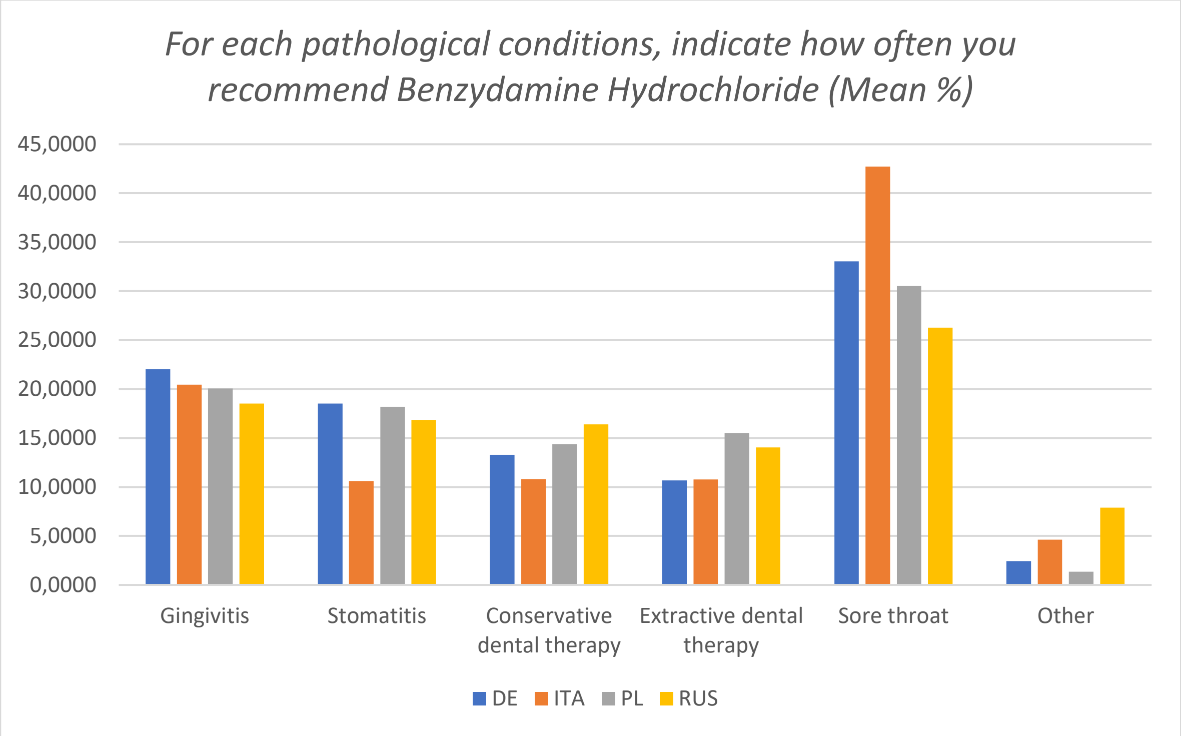

Table 4.10 - For which of the following symptoms reported by the customer do you recommend Benzydamine Hydrochloride (Tantum Verde)? (indicate in percentage)

Country: Germany	Ache	Itchy throat	Difficulty in swallowing	Dry throat	Burning of the mouth	Redness of the throat	Tonsillitis
Min.	0	0	0	0	0	0	0
1st Qu.	0	0	0	0	0	0	0
Median	5	0	0	0	4.50	10	10
Mean	8.341	3.537	5.280	2.341	5.841	9.415	10.768
3rd Qu.	10	5	7	2.25	10	20	20
Max.	60	22	50	30	30	50	40
NA's	18	18	18	18	18	18	18

	Distorted voice	Cough	Ulcerative lesions or canker sores	Halitosis	Temperature	All symptoms due to extraction therapy	Gingivitis
Min.	0	0	0	0	0	0	0
1st Qu.	0	0	3.25	0	0	0	0
Median	0	4	10	0	0	10	10
Mean	1.866	8.195	11.671	5.354	1.366	9.829	16.195
3rd Qu.	0	17.50	20	10	0	15	27.50
Max.	20	50	50	30	30	50	100
NA's	18	18	18	18	18	18	18

Country: Italy	Ache	Itchy throat	Difficulty in swallowing	Dry throat	Burning of the mouth	Redness of the throat	Tonsillitis
Min.	0	0	0	0	0	0	0
1st Qu.	10	6.25	2.25	0	0	5	3
Median	17.5	15	5	5	3	10	5
Mean	18.400	16.000	4.500	4.000	3.814	11.571	5.329
3rd Qu.	30	25	5	5	5	15	8.75
Max.	50	45	20	20	40	50	20
NA's	30	30	30	30	30	30	30

	Distorted voice	Cough	Ulcerative lesions or canker sores	Halitosis	Temperature	All symptoms due to extraction therapy	Gingivitis
Min.	0	0	0	0	0	0	0
1st Qu.	0	3	2	0	0	2	5
Median	3	10	5	0	2	5	5
Mean	2.600	11.229	5.586	1.714	2.300	5.657	7.300
3rd Qu.	5	15	10	3	5	10	10
Max.	10	30	30	20	10	30	30
NA's	30	30	30	30	30	30	30

Country: Poland	Ache	Itchy throat	Difficulty in swallowing	Dry throat	Burning of the mouth	Redness of the throat	Tonsillitis
Min.	0	0	0	0	0	0	0
1st Qu.	0	0	0	0	0	5	5
Median	5	5	5	3.5	5	8.5	10
Mean	8.59	4.897	5.551	5.385	5.487	8.474	8.423
3rd Qu.	15	5.75	10	5	10	10	10
Max.	30	20	30	70	30	50	40
NA's	23	23	23	23	23	23	23

	Distorted voice	Cough	Ulcerative lesions or canker sores	Halitosis	Temperature	All symptoms due to extraction therapy	Gingivitis
Min.	0	0	0	0	0	0	0
1st Qu.	0	5	0	0	0	5	5
Median	0	10	5	1	0	10	10
Mean	2.821	12.256	7.628	4.487	2.41	14.295	9.295
3rd Qu.	5	20	10	5	4.25	20	10
Max.	20	60	50	20	20	60	50
NA's	23	23	23	23	23	23	23

Country: Russia	Ache	Itchy throat	Difficulty in swallowing	Dry throat	Burning of the mouth	Redness of the throat	Tonsillitis
Min.	0	0	0	0	0	0	0
1st Qu.	5	5	0.5	0	0	3	5
Median	20	10	5	4	5	5	7
Mean	16.192	8.795	5.769	3.590	5.654	7.679	7.410
3rd Qu.	25	15	9.75	5	7	10	10
Max.	40	25	25	20	36	30	20
NA's	22	22	22	22	22	22	22

	Distorted voice	Cough	Ulcerative lesions or canker sores	Halitosis	Temperature	All symptoms due to extraction therapy	Gingivitis
Min.	0	0	0	0	0	0	0
1st Qu.	0	2.25	2	0	0	5	2.25
Median	2	8	5	5	0.5	6.5	5
Mean	3.731	10.218	7.603	4.654	4.000	8.295	6.410
3rd Qu.	4	15	10	7	4	10	10
Max.	25	50	30	40	80	35	30
NA's	22	22	22	22	22	22	22

Table 4.11 - Which formulation of Benzydamine Hydrochloride (Tantum Verde) do you recommend the most? (indicate the percentage)

Country: Germany	Mouthwash 0.15	Spray 0.15	Spray 0.30
Min.	10	10	10
1st Qu.	25	30	20
Median	30	40	30
Mean	31.951	37.866	30.183
3rd Qu.	40	50	40
Max.	60	80	60
NA's	18	18	18

Country: Italy	Mouthwash 0.15	Spray 0.15	Spray 0.30
Min.	10	5	10
1st Qu.	15	15	20
Median	40	20	25
Mean	37.286	21.143	32.714
3rd Qu.	55	25	55
Max.	80	70	70
NA's	30	30	30

Country: Poland	Mouthwash 0.15	Spray 0.15	Spray 0.30
Min.	0	0	0
1st Qu.	20	20	16.25
Median	30	20	20
Mean	34.654	23.692	25.385
3rd Qu.	50	30	30
Max.	100	50	100
NA's	23	23	23

Country: Russia	Mouthwash 0.15	Spray 0.15	Spray 0.30
Min.	0	10	10
1st Qu.	15	20	20
Median	20	25	30
Mean	24.064	25.244	30.551
3rd Qu.	30	30	43
Max.	60	50	70
NA's	22	22	22

Table 4.12 - Do you usually provide information on the dosage and duration of treatment with Benzydamine Hydrochloride (Tantum Verde)?

Value	Country	n	n/N
Yes	DE	75	0.915
No	DE	7	0.100
Yes	ITA	65	0.833
No	ITA	5	0.064
Yes	PL	57	0.695
No	PL	21	0.300
Yes	RUS	66	0.846
No	RUS	12	0.154

Table 4.13 - Which are the main characteristics that make you recommend Benzydamine Hydrochloride (Tantum Verde)? (1=Not very important, 5=Very important)

Preference	Country	Anesthetics	Analgesics	Anti-inflammatory	Antiseptics
1Not very important	DE	0.073	0.122	0.073	0.085
2	DE	0.243	0.143	0.057	0.014
3	DE	0.256	0.269	0.346	0.192
4	DE	0.308	0.231	0.346	0.551
5 Very important	DE	0.183	0.280	0.220	0.195
Average score	DE	3.305	3.415	3.573	3.732
1	ITA	0.014	0.071	0.100	0.029
2	ITA	0.077	0.103	0.077	0.154
3	ITA	0.333	0.179	0.064	0.141
4	ITA	0.183	0.220	0.159	0.341
5	ITA	0.314	0.357	0.557	0.243
Average score	ITA	3.729	3.714	4.014	3.657
1	PL	0.038	0.077	0.013	0.038
2	PL	0.103	0.167	0.090	0.026
3	PL	0.122	0.171	0.293	0.073
4	PL	0.400	0.386	0.257	0.486
5	PL	0.372	0.231	0.359	0.423
Average score	PL	3.923	3.487	3.833	4.179
1	RUS	0.013	0.000	0.013	0.038
2	RUS	0.061	0.073	0.073	0.037
3	RUS	0.086	0.100	0.057	0.214
4	RUS	0.282	0.308	0.295	0.295
5	RUS	0.564	0.526	0.564	0.436
Average score	RUS	4.321	4.282	4.321	4.051

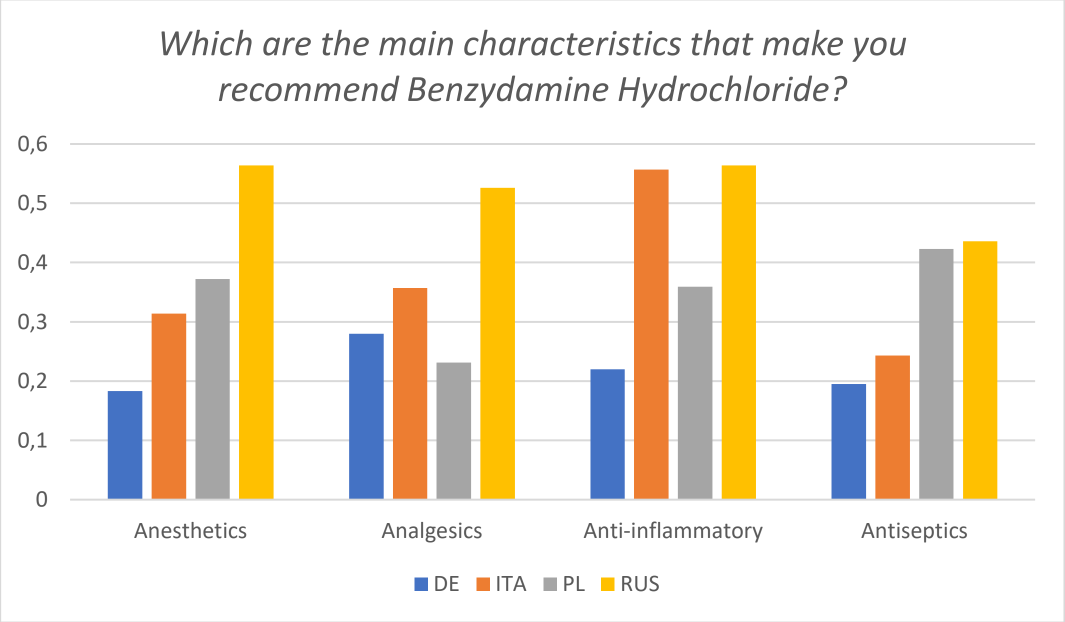

Table 4.14 - What is the percentage of consumers that ask you for advice / suggestions on Benzydamine Hydrochloride (Tantum Verde) and its use?

Country: Germany	Yes	No
Min.	0	0
1st Qu.	30	30
Median	55	45
Mean	52.95	47.05
3rd Qu.	70	70
Max.	100	100

Country: Italy	Yes	No
Min.	10	20
1st Qu.	40	45
Median	50	50
Mean	46.70	53.30
3rd Qu.	55	60
Max.	80	90

Country: Poland	Yes	No
Min.	0	10
1st Qu.	30	40
Median	50	50
Mean	46.19	53.81
3rd Qu.	60	70
Max.	90	100

Country: Russia	Yes	No
Min.	0	10
1st Qu.	30	35
Median	50	50
Mean	47.74	52.26
3rd Qu.	65	70
Max.	90	100

Table 4.15 - In your experience, out of the TOTAL of customers who request / purchase Benzydamine Hydrochloride (Tantum Verde) in what percentage they choose it:

Country: Germany	Self management	On medical advice	On pediatrician advice	On pharmacist advice
Min.	0	0	0	0
1st Qu.	30	15	10	10
Median	30	25	20	20
Mean	36.85	25.25	16.35	21.55
3rd Qu.	45	30	20	26.25
Max.	100	50	50	100

Country: Italy	Self management	On medical advice	On pediatrician advice	On pharmacist advice
Min.	10	10	5	0
1st Qu.	25	20	10	5
Median	37.5	25	25	10
Mean	40.15	25.85	22.65	11.35
3rd Qu.	55	30	30	15
Max.	80	50	45	50

Country: Poland	Self management	On medical advice	On pediatrician advice	On pharmacist advice
Min.	10	0	0	0
1st Qu.	25	12	10	10
Median	30	20	15	20
Mean	40.89	22.40	16.47	20.25
3rd Qu.	50	30	25	30
Max.	100	60	40	60

Country: Russia	Self management	On medical advice	On pediatrician advice	On pharmacist advice
Min.	0	0	0	0
1st Qu.	25	20	10	10
Median	40	20	19	10
Mean	42.2	23.5	20.48	13.82
3rd Qu.	60	30	30	15
Max.	90	60	70	60

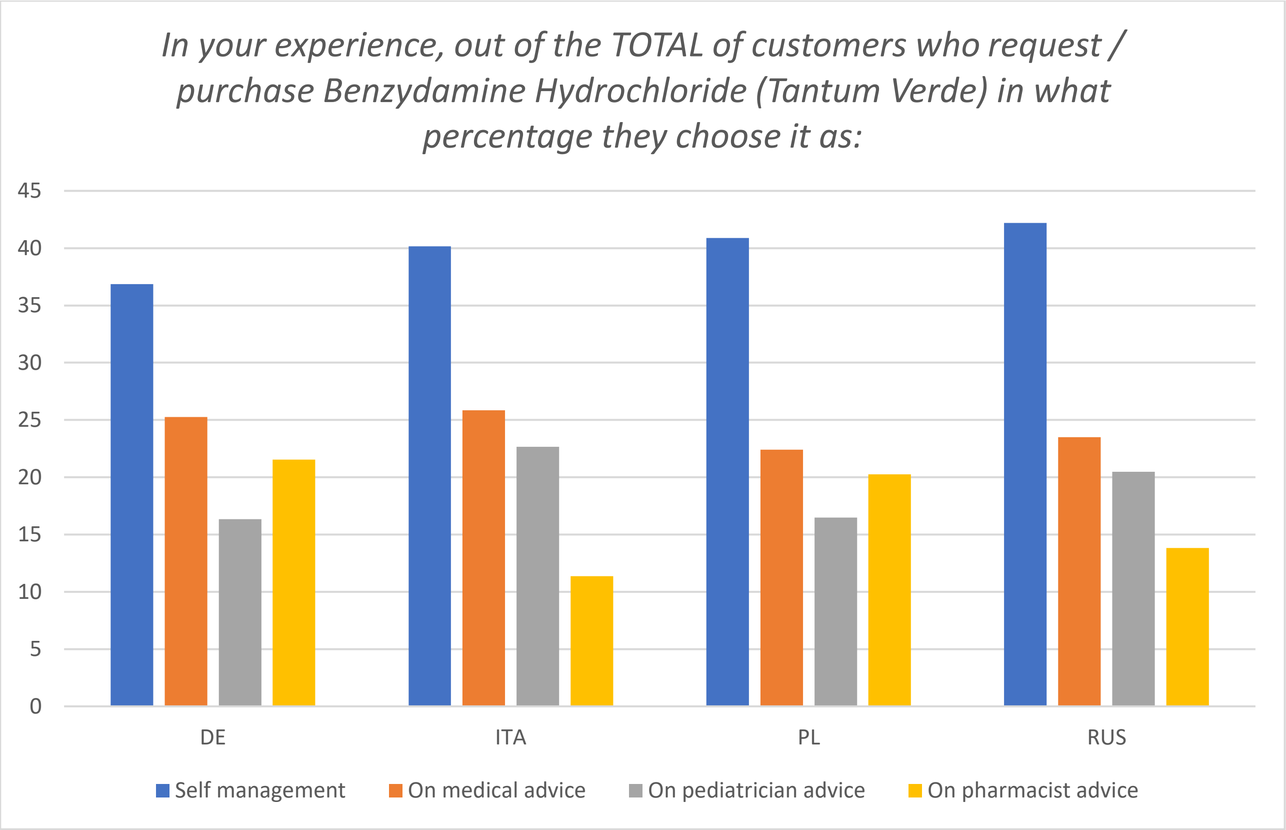
In your experience, out of the TOTAL of customers who request / purchase Benzydamine Hydrochloride (Tantum Verde) in what percentage they choose it as:
45
40
35
30
25
20
15
10
5
0
DE
ITA
PL
RUS
Self management
On medical advice
On pediatrician advice
On pharmacist advice

4.2 General practitioners

Table 4.16 - Based on your knowledge, are the following active ingredients usable for the topical medication of sore throat and inflammatory/irritative conditions of the mouth such as gingivitis or stomatitis?

	Country	Ketoprofene	Flurbiprofene	Ambroxolo Cloridrato	Benzyl alcohol - sodium benzoate	Benzydamine hydrochloride	Natural extracts
Yes	DE	0.73	0.74	0.74	0.74	0.87	0.52
No	DE	0.21	0.23	0.24	0.23	0.10	0.33
Don't know	DE	0.06	0.03	0.02	0.03	0.03	0.15
Yes	ITA	0.74	0.77	0.82	0.76	0.89	0.49
No	ITA	0.22	0.21	0.16	0.19	0.09	0.36
Don't know	ITA	0.03	0.01	0.01	0.04	0.01	0.15
Yes	PL	0.75	0.87	0.94	0.94	0.92	0.67
No	PL	0.19	0.09	0.04	0.05	0.07	0.24
Don't know	PL	0.06	0.04	0.02	0.01	0.01	0.09
Yes	RUS	0.78	0.83	0.92	0.97	1.01	0.83
No	RUS	0.19	0.18	0.08	0.04	0.00	0.15
Don't know	RUS	0.04	0.00	0.01	0.00	0.00	0.03

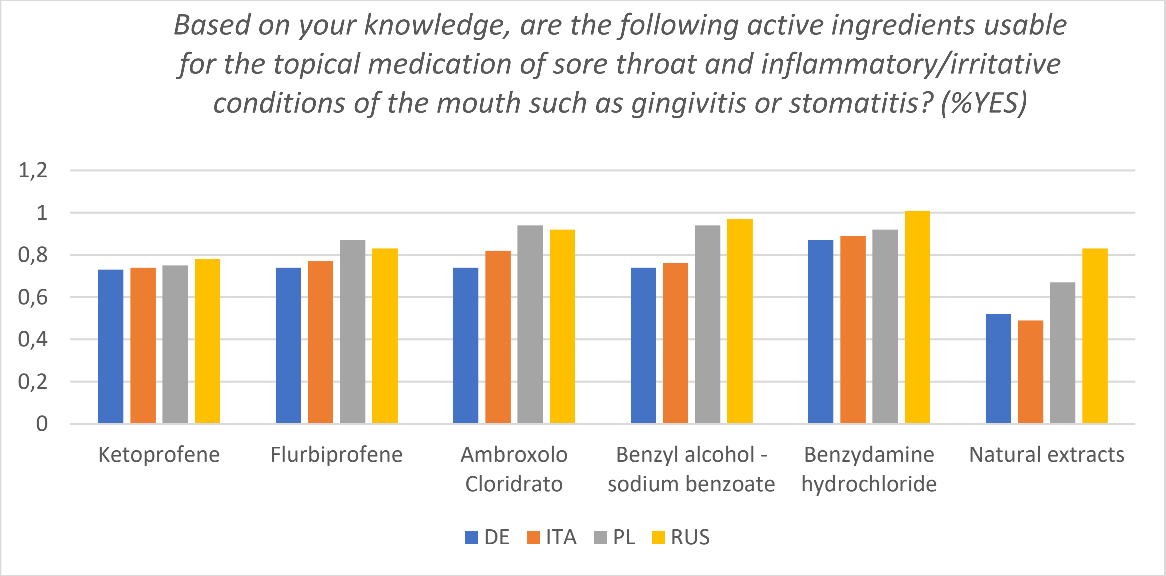

Table 4.17 - In general, which active ingredients prescribed / recommended for the topical treatment of sore throat symptoms and various inflammatory / irritative conditions of the oral cavity such as gingivitis, stomatitis? (For each answer, tick Yes or No)

Value	Country	Ketoprofene	Flurbiprofene	Ambroxolo Cloridrato	Benzyl alcohol - sodium benzoate	Benzydamine hydrochloride	Natural extracts
Yes	DE	0.70	0.71	0.74	0.81	0.73	0.40
No	DE	0.30	0.29	0.26	0.19	0.27	0.60
Yes	ITA	0.63	0.67	0.72	0.72	0.89	0.46
No	ITA	0.37	0.33	0.28	0.28	0.11	0.54
Yes	PL	0.69	0.86	0.91	0.94	0.72	0.42
No	PL	0.31	0.14	0.09	0.06	0.28	0.58
Yes	RUS	0.78	0.81	0.89	0.88	0.90	0.69
No	RUS	0.23	0.20	0.12	0.13	0.11	0.32

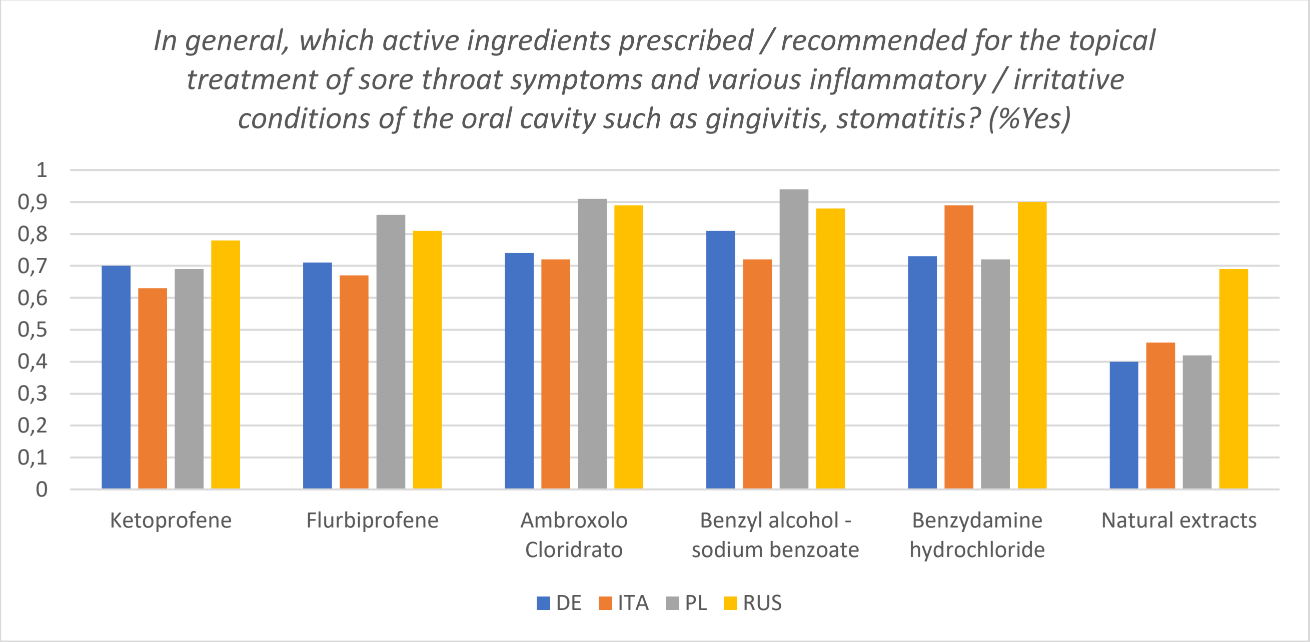

Table 4.18 – Assign an order of preference to each of the following active ingredients for the topical treatment of sore throat symptoms and various inflammatory / irritative conditions of the oral cavity such as gingivitis, stomatitis and following extractive therapies (1 = most preferred, 7 = least preferred)

Country: Germany	Ketoprofene	Flurbiprofene Ambroxolo	Cloridrato	Alcool Benzilico	Benzidamina Cloridrato	Natural extracts	Others
Min.	1	1	1	1	1	1	2
1st Qu.	2	2	2	2	2	6	7
Median	4	3	3	3	4	6	7
Avs Score	3.35	3.19	3.23	3.02	3.48	5.08	6.60
3rd Qu.	5	4	5	4	5	6	7
Max.	6	6	7	6	7	7	7
NA's	1	2	3	3	3	3	42

Country: Italy	Ketoprofene	Flurbiprofene Ambroxolo	Cloridrato	Alcool Benzilico	Benzidamina Cloridrato	Natural extracts	Others
Min.	1	1	1	1	1	1	2
1st Qu.	2	2	2	2	2	4.75	7
Median	2.5	3	3	4	3	6	7
Mean	2.96	3.07	3.08	3.78	3.33	5.14	6.26
3rd Qu.	5	4	4	5	5	6	7
Max.	6	6	7	7	7	7	7
NA's	1	1	2	3	4	8	57

Country: Poland	Ketoprofene	Flurbiprofene Ambroxolo	Cloridrato	Alcool Benzilico	Benzidamina Cloridrato	Natural extracts	Others
Min.	1	1	1	1	1	1	1
1st Qu.	2	2	2	2	1	5	7
Median	3	3	3	3	4	6	7
Mean	3.30	3.21	3.29	3.15	3.34	4.88	6.86
3rd Qu.	4	4	4	4	5	6	7
Max.	6	6	7	7	6	7	7
NA's	1	1	1	1	1	1	21

Country: Russia	Ketoprofene	Flurbiprofene Ambroxolo	Cloridrato	Alcool Benzilico	Benzidamina Cloridrato	Natural extracts	Others
Min.	1	1	1	1	1	1	2
1st Qu.	1	1	3	3	3	5	7
Median	2	3	3	4	4	6	7
Mean	2.38	2.87	3.44	3.50	3.95	5.03	6.78
3rd Qu.	3	4	4	4	5	6	7
Max.	6	7	7	6	6	7	7
NA's	1	1	1	1	1	1	55

Table 4.19 - Your therapeutic approach involves

Value	Country	n	n/N
Start with topical medication	DE	28	0.431
Start with topical medication and move on to systemic	DE	17	0.386
No preference	DE	20	0.253
Start with topical medication	IT	14	0.286
Start with topical medication and move on to systemic	IT	26	0.400
No preference	IT	4	0.091
Start with topical medication	PL	30	0.380
Start with topical medication and move on to systemic	PL	20	0.408
No preference	PL	29	0.446
Start with topical medication	RUS	23	0.523
Start with topical medication and move on to systemic	RUS	23	0.291
No preference	RUS	3	0.061

Table 4.20 - Which of the following formulations do you prescribe/recommend for the treatment of sore throat symptoms in adults? Assign an order of preference to each (1 = most preferred, 4 = least preferred)

Preference	Country	Spray	Hard candy	Mouthwash	Soft tabs
1 Highest	DE	0.66	0.24	0.06	0.04
2	DE	0.10	0.34	0.42	0.14
3	DE	0.17	0.37	0.30	0.16
4 Lowest	DE	0.07	0.05	0.22	0.65
Average score	DE	1.65	2.23	2.68	3.44
1 Highest	IT	0.48	0.37	0.11	0.04
2	IT	0.14	0.16	0.56	0.14
3	IT	0.35	0.45	0.17	0.03
4 Lowest	IT	0.03	0.02	0.16	0.78
Average score	IT	1.93	2.12	2.38	3.57
1 Highest	PL	0.69	0.23	0.07	0.01
2	PL	0.04	0.38	0.38	0.20
3	PL	0.20	0.38	0.16	0.26
4 Lowest	PL	0.07	0.01	0.39	0.53
Average score	PL	1.65	2.17	2.87	3.31
1 Highest	RUS	0.44	0.31	0.18	0.08
2	RUS	0.22	0.22	0.30	0.27
3	RUS	0.25	0.32	0.27	0.17
4 Lowest	RUS	0.10	0.16	0.26	0.49
Average score	RUS	2.01	2.33	2.60	3.06

Table 4.21 - Which of the following formulations do you prescribe / recommend for the treatment of sore throat symptoms in children? Assign an order of preference to each (1 = most preferred, 3 = least preferred)

Preference	Country	Benzidamina Cloridrato	Diclorobenzil Alcool 2 mg Sodio Benzoato 20 mg	Natural extracts
1 Highest	DE	0.38	0.40	0.21
2	DE	0.46	0.47	0.06
3	DE	0.16	0.12	0.70
4 Lowest	DE	0.00	0.01	0.03
Average score	DE	1.78	1.74	2.55
1 Highest	IT	0.60	0.33	0.06
2	IT	0.30	0.45	0.25
3	IT	0.09	0.22	0.69
4 Lowest	IT	0.00	0.00	0.00
Average score	IT	1.48	1.89	2.63
1 Highest	PL	0.28	0.45	0.27
2	PL	0.48	0.43	0.09
3	PL	0.24	0.12	0.64
4 Lowest	PL	0.00	0.00	0.00
Average score	PL	1.96	1.67	2.37
1 Highest	RUS	0.32	0.45	0.24
2	RUS	0.37	0.42	0.22
3	RUS	0.32	0.14	0.55
4 Lowest	RUS	0.00	0.00	0.00
Average score	RUS	2.00	1.69	2.31

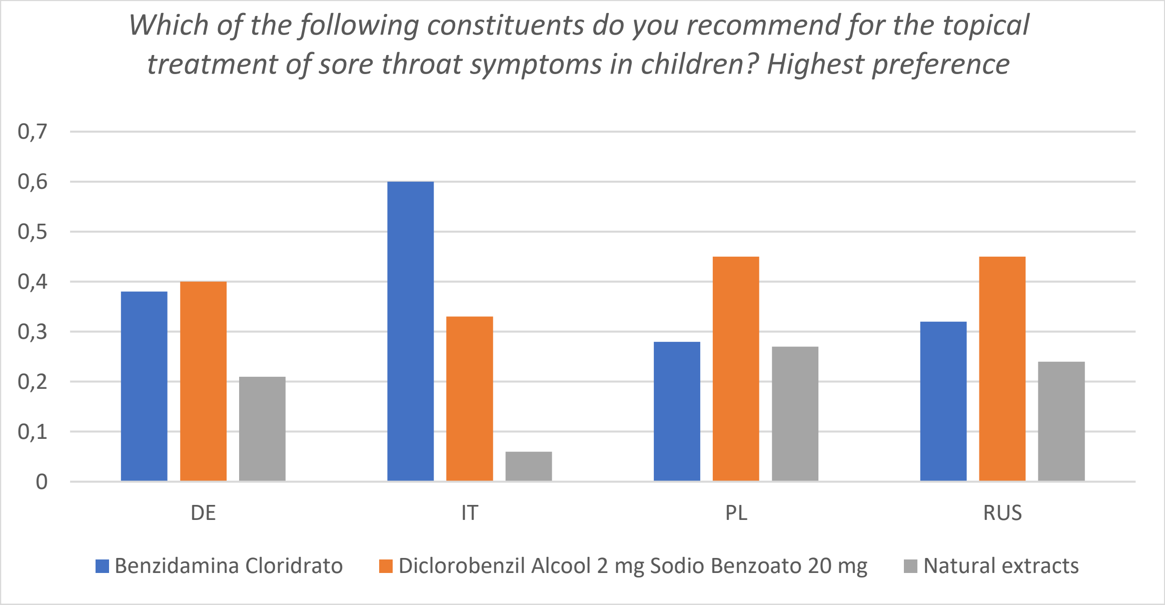

Table 4.22 - Which of the following formulations do you prescribe / recommend for the treatment of sore throat symptoms in children? Assign an order of preference to each (1 = most preferred formulation, 3 = least preferred formulation)

Preference	Country	Spray	Hard candy	Soft tabs
1 Highest	DE	0.50	0.20	0.27
2	DE	0.34	0.45	0.18
3 Lowest	DE	0.13	0.32	0.52
Average score	DE	1.62	2.12	2.26
1 Highest	IT	0.43	0.23	0.34
2	IT	0.40	0.42	0.18
3 Lowest	IT	0.17	0.35	0.48
Average score	IT	1.74	2.12	2.14
1 Highest	PL	0.32	0.25	0.43
2	PL	0.44	0.36	0.20
3 Lowest	PL	0.24	0.39	0.37
Average score	PL	1.92	2.14	1.94
1 Highest	RUS	0.28	0.38	0.35
2	RUS	0.32	0.39	0.30
3 Lowest	RUS	0.41	0.24	0.36
Average score	RUS	2.13	1.86	2.01

Table 4.23 - Do you prescribe / recommend Benzydamine hydrochloride (Tantum Verde)?

Value	Country	n/N
Yes	DE	0.71
No	DE	0.29
Yes	IT	0.77
No	IT	0.23
Yes	PL	0.67
No	PL	0.33
Yes	RUS	0.73
No	RUS	0.28

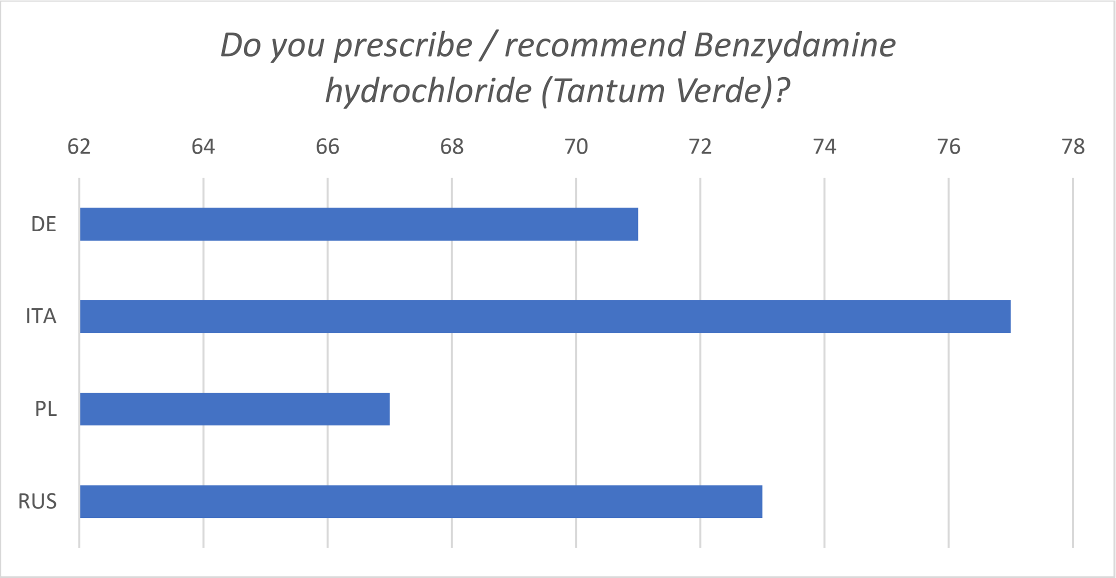

The following questions were administered only to doctors who recommend/prescribe Tantum Verde

Table 4.24 - How often do you prescribe / recommend Benzydamine hydrochloride (Tantum verde)?

Values	Country	n	n/N
0 –| 20%	DE	17	0.17
20 –| 50%	DE	18	0.18
50 –| 70%	DE	27	0.27
70 -|100%	DE	9	0.09
NA	DE	29	0.29
0 –| 20%	IT	16	0.16
20 –| 50%	IT	26	0.26
50 –| 70%	IT	30	0.30
70 -|100%	IT	5	0.05
NA	IT	23	0.23
0 –| 20%	PL	18	0.18
20 –| 50%	PL	27	0.27
50 –| 70%	PL	17	0.17
70 -|100%	PL	5	0.05
NA	PL	33	0.33
0 –| 20%	RUS	13	0.13
20 –| 50%	RUS	24	0.24
50 –| 70%	RUS	34	0.34
70 -|100%	RUS	2	0.02
NA	RUS	28	0.28

Table 4.25 - For each of the following pathological conditions, indicate the percentage of times you prescribe / recommend Benzydamine hydrochloride (Tantum Verde)

Country: Germany	Gingivitis	Stomatitis	Conservative dental therapy	Extractive dental therapy	Sore throat	Other
Min.	0	0	0	0	0	0
1st Qu.	10	12.50	10	0	5	0
Median	20	20	20	10	20	0
Mean	18.85	20.18	18.70	13.47	25.00	3.80
3rd Qu.	27.50	30	25	20	40	0
Max.	100	50	100	50	100	50
NA's	29	29	29	29	29	29

Country: Italy	Gingivitis	Stomatitis	Conservative dental therapy	Extractive dental therapy	Sore throat	Other
Min.	0	0	0	0	0	0
1st Qu.	10	10	10	10	10	0
Median	20	20	20	20	20	0
Mean	20.07	14.94	18.83	17.01	26.69	2.47
3rd Qu.	25	20	20	20	40	0
Max.	60	50	55	55	100	20
NA's	23	23	23	23	23	23

Country: Poland	Gingivitis	Stomatitis	Conservative dental therapy	Extractive dental therapy	Sore throat	Other
Min.	0	0	0	0	0	0
1st Qu.	10	10	7.5	10	20	0
Median	20	20	15	20	25	0
Mean	15.97	17.76	13.81	17.99	33.81	0.67
3rd Qu.	20	25	20	20	40	0
Max.	80	50	40	100	100	10
NA's	33	33	33	33	33	33

Country: Russia	Gingivitis	Stomatitis	Conservative dental therapy	Extractive dental therapy	Sore throat	Other
Min.	0	0	0	0	0	0
1st Qu.	15	10	10	10	15	0
Median	23	15	10	10	20	10
Mean	21.80	14.33	13.30	14.96	25.07	10.55
3rd Qu.	25	20	15	20	35	15
Max.	40	35	50	50	70	60
NA's	28	28	28	28	28	28

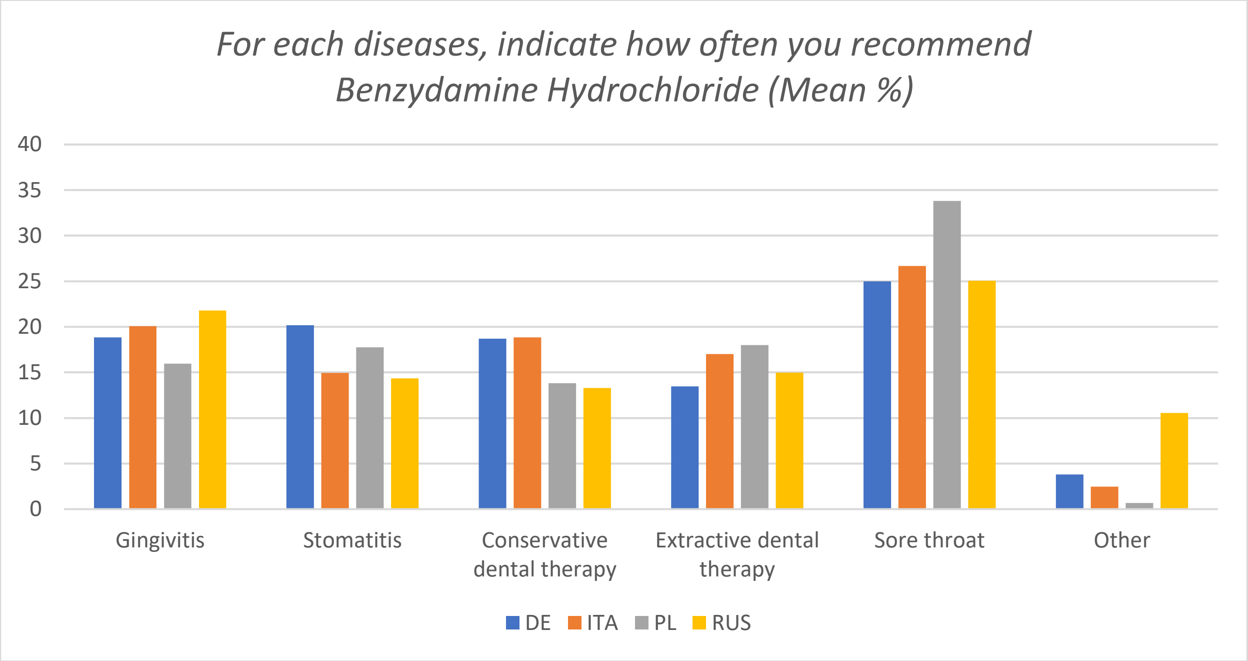

Table 4.26 - For each of the following patient-reported symptoms, indicate the percentage of times you prescribe / recommend Benzydamine hydrochloride (Tantum Verde)

Country: Germany	Ache	Itchy and itchy throat	Difficulty in swallowing	Dry throat	Burning of the mouth	Redness of the throat
Min.	0	0	0	0	0	0
1st Qu.	0	0	0	0	0	0
Median	0	0	0	0	0	5
Mean	8.48	3.85	4.70	1.97	7.76	8.80
3rd Qu.	20	10	10	0	10	10
Max.	40	20	30	20	50	40
NA's	29	29	29	29	29	29

	Distorted voice	Cough	Ulcerative lesions or canker sores	Halitosis	Temperature	All symptoms due to extraction therapy
Min.	0	0	0	0	0	0
1st Qu.	0	0	0	0	0	0
Median	0	10	10	0	0	15
Mean	3.38	11.76	16.17	5.42	0.78	18.21
3rd Qu.	0	20	20	10	0	30
Max.	50	80	100	50	10	100
NA's	29	29	29	29	29	29

Country: Italy	Ache	Itchy and itchy throat	Difficulty in swallowing	Dry throat	Burning of the mouth	Redness of the throat	Tonsillitis
Min.	0	0	0	0	0	0	0
1st Qu.	0	5	5	0	5	5	0
Median	10	10	5	5	10	10	10
Mean	11.40	10.46	7.66	6.10	11.82	9.78	7.04
3rd Qu.	20	15	10	10	20	10	10
Max.	90	50	50	50	50	30	20
NA's	23	23	23	23	23	23	23

	Distorted voice	Cough	Ulcerative lesions or canker sores	Halitosis	Temperature	All symptoms due to extraction therapy
Min.	0	0	0	0	0	0
1st Qu.	0	0	5	0	0	0
Median	0	5	10	0	0	10
Mean	3.52	8.60	10.10	4.17	1.22	8.13
3rd Qu.	5	10	15	10	0	10
Max.	75	50	50	20	15	30
NA's	23	23	23	23	23	23

Country: Poland	Ache	Itchy and itchy throat	Difficulty in swallowing	Dry throat	Burning of the mouth	Redness of the throat	Tonsillitis
Min.	0	0	0	0	0	0	0
1st Qu.	0	0	0	0	0	0	5
Median	5	5	2	0	5	5	10
Mean	4.55	5.55	3.63	3.43	7.31	7.39	12.70
3rd Qu.	10	5	5	5	10	10	10
Max.	20	50	15	60	50	50	100
NA's	33	33	33	33	33	33	33

	Distorted voice	Cough	Ulcerative lesions or canker sores	Halitosis	Temperature	All symptoms due to extraction therapy
Min.	0	0	0	0	0	0
1st Qu.	0	5	0	0	0	10
Median	0	10	10	0	0	20
Mean	3.12	13.81	10.22	5.60	1.75	20.94
3rd Qu.	5	20	17.5	10	0	30
Max.	20	50	50	30	20	100
NA's	33	33	33	33	33	33

Country: Russia	Ache	Itchy and itchy throat	Difficulty in swallowing	Dry throat	Burning of the mouth	Redness of the throat	Tonsillitis
Min.	0	0	0	0	0	0	0
1st Qu.	10	5	5	0	0	3	5
Median	22	10	5	5	5	5	7
Mean	18.1	10.5	6.5	4.0	6.0	8.2	7.3
3rd Qu.	25	17	10	5	7	10	10
Max.	40	20	20	20	30	30	20
NA's	28	28	28	28	28	28	28

	Distorted voice	Cough	Ulcerative lesions or canker sores	Halitosis	Temperature	All symptoms due to extraction therapy
Min.	0	0	0	0	0	0
1st Qu.	0	4	2	0	0	5
Median	2	8	5	5	1	10
Mean	2.5	9.2	9.0	4.8	2.7	11.2
3rd Qu.	4	15	10	10	5	10
Max.	20	40	50	20	10	80
NA's	28	28	28	28	28	28

Table 4.27 - For each of the following formulations of Benzydamine hydrochloride (Tantum Verde), indicate the percentage of times you prescribe / recommend it

Country: Germany	Mouthwash 0.15	Spray 0.15	Spray 0.30
Min.	0	0	0
1st Qu.	30	30	10
Median	40	30	20
Mean	36.48	37.89	25.63
3rd Qu.	50	45	40
Max.	100	100	80
NA's	29	29	29

Country: Italy	Mouthwash 0.15	Spray 0.15	Spray 0.30
Min.	0	5	0
1st Qu.	30	20	15
Median	50	20	20
Mean	43.83	22.21	21.43
3rd Qu.	50	30	30
Max.	90	50	50
NA's	23	23	23

Country: Poland	Mouthwash 0.15	Spray 0.15	Spray 0.30
Min.	0	0	0
1st Qu.	20	10	15
Median	30	20	20
Mean	40.22	17.91	25.49
3rd Qu.	50	20	30
Max.	100	50	90
NA's	33	33	33

Country: Russia	Mouthwash 0.15	Spray 0.15	Spray 0.30
Min.	0	0	0
1st Qu.	20	15	20
Median	25	20	30
Mean	28.92	22.21	26.15
3rd Qu.	35	25	30
Max.	80	40	50
NA's	28	28	28

Table 4.28 - Do you usually provide information on the posology and duration of treatment with Benzydamine hydrochloride (Tantum Verde)?

Value	Country	n	n/N
Yes	DE	61	0.61
No	DE	39	0.39
NA	DE	0	0.00
Yes	IT	62	0.61
No	IT	15	0.15
NA	IT	23	0.23
Yes	PL	44	0.44
No	PL	23	0.23
NA	PL	33	0.33
Yes	RUS	61	0.61
No	RUS	12	0.12
NA	RUS	28	0.28

Table 4.29 - Which are the main characteristics that make you prescribe / recommend Benzydamine hydrochloride (Tantum Verde)? (1=Not very important, 5=Very important)

Preference	Country	Anesthetics	Analgesics	Anti-inflammatory	Antiseptics
1 Not very important	DE	0.14	0.07	0.05	0.03
2	DE	0.13	0.19	0.08	0.03
3	DE	0.14	0.18	0.29	0.13
4	DE	0.19	0.18	0.19	0.39
5 Very important	DE	0.11	0.09	0.10	0.13
Average score	DE	3.00	3.04	3.30	3.79
NA	DE	0.29	0.29	0.29	0.29
1	IT	0.16	0.12	0.08	0.09
2	IT	0.13	0.13	0.14	0.12
3	IT	0.17	0.19	0.16	0.15
4	IT	0.22	0.23	0.10	0.33
5	IT	0.09	0.10	0.29	0.08
Average score	IT	2.94	3.08	3.49	3.25
NA	IT	0.23	0.23	0.23	0.23
1	PL	0.05	0.03	0.01	0.00
2	PL	0.05	0.06	0.10	0.00
3	PL	0.09	0.23	0.16	0.09
4	PL	0.21	0.17	0.21	0.25
5	PL	0.27	0.18	0.19	0.33
Average score	PL	3.90	3.61	3.70	4.36
NA	PL	0.33	0.33	0.33	0.33
1	RUS	0.01	0.00	0.01	0.02
2	RUS	0.07	0.03	0.06	0.04
3	RUS	0.01	0.05	0.05	0.12
4	RUS	0.17	0.21	0.14	0.21
5	RUS	0.47	0.44	0.47	0.34
Average score	RUS	4.40	4.45	4.37	4.11
NA	RUS	0.28	0.28	0.28	0.28

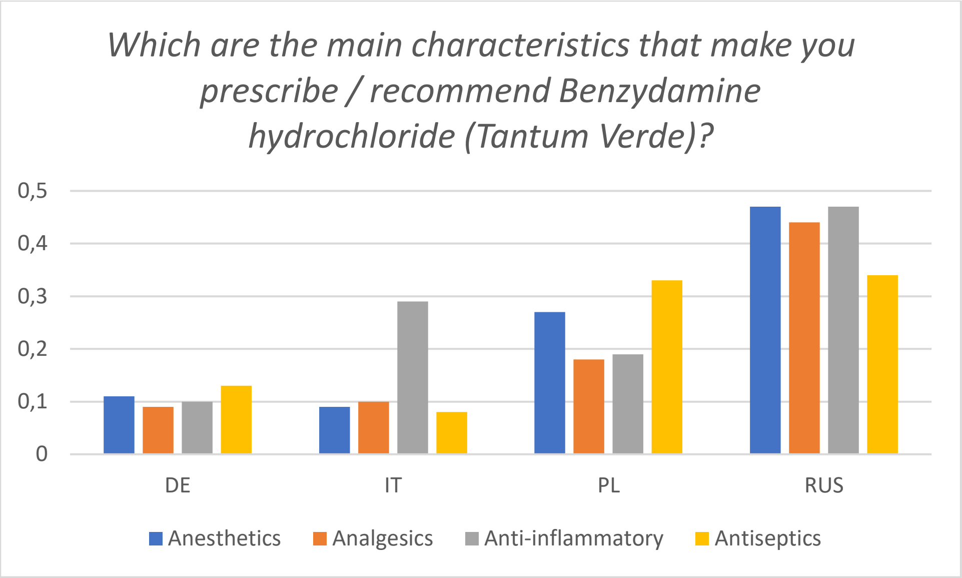

Table 4.30 - Do you prescribe / recommend Benzydamine hydrochloride (Tantum Verde) in combination with other drugs?

Value	Country	n	n/N
Yes	DE	50	0.50
No	DE	21	0.21
NA	DE	29	0.29
Yes	IT	54	0.54
No	IT	23	0.23
NA	IT	23	0.23
Yes	PL	41	0.41
No	PL	26	0.26
NA	PL	33	0.33
Yes	RUS	58	0.58
No	RUS	15	0.15
NA	RUS	28	0.28

Table 4.31 - Do you prescribe / recommend Benzydamine hydrochloride (Tantum Verde) for children?

Value	Country	n	n/N
Yes	DE	52	0.52
No	DE	19	0.19
NA	DE	29	0.29
Yes	IT	61	0.60
No	IT	16	0.16
NA	IT	23	0.23
Yes	PL	53	0.53
No	PL	14	0.14
NA	PL	33	0.33
Yes	RUS	67	0.67
No	RUS	6	0.06
NA	RUS	28	0.28

DEMOGRAPHICS

Pharmacists - Registry

Gender

Value	Country	n	n/N
Female	DE	50	0.500
Male	DE	50	0.500
Female	ITA	44	0.436
Male	ITA	56	0.560
Female	PL	39	0.390
Male	PL	62	0.620
Female	RUS	49	0.485
Male	RUS	51	0.510

Age

	DE	ITA	PL	RUS
Min.	31	27	22	28
1st Qu.	49	35	50	38.75
Median	55	42	54	49.5
Mean	53.86	44.00	52.15	48.61
3rd Qu.	61	52.25	58	59
Max.	71	66	61	70

Customer base

Value	DE n/N	ITA n/N	PL n/N	RUS n/N
0-|300	0.070	0.110	0.120	0.140
300 -| 700	0.750	0.740	0.610	0.640
700 - 1000	0.209	0.151	0.116	0.233
1000+	0.000	0.020	0.030	0.010

General practitioners - Registry by country

Gender distribution

Value	Country	n	n/N
Female	DE	46	0.46
Male	DE	54	0.54
Female	IT	41	0.41
Male	IT	59	0.59
Female	PL	35	0.35
Male	PL	65	0.65
Female	RUS	50	0.50
Male	RUS	50	0.50

Age

	Germany	Italy	Poland	Russia
Min.	30	31	37	29
1st Qu.	53	44	47	39
Median	56	52	53	52
Mean	56.81	51.00	52.09	49.91
3rd Qu.	63	59	58	60
Max.	71	67	69	69

Customer base

	Germany	Italy	Poland	Russia
Min.	300	350	55	200
1st Qu.	1000	950	1000	850
Median	1200	1100	1300	1000
Mean	1162.85	1097.78	1219.69	1004.16
3rd Qu.	1300	1250	1465	1200
Max.	1580	1600	1560	1520
NA's	0	0	2	0

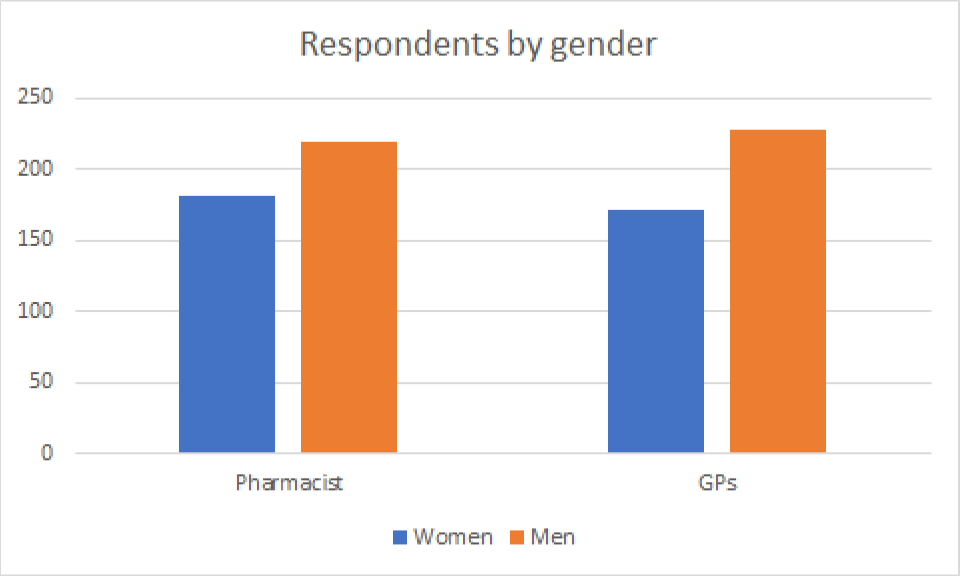

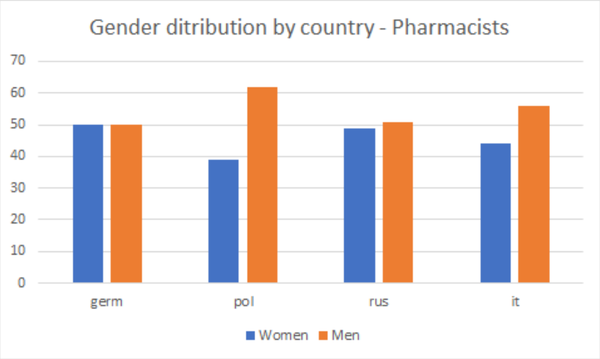

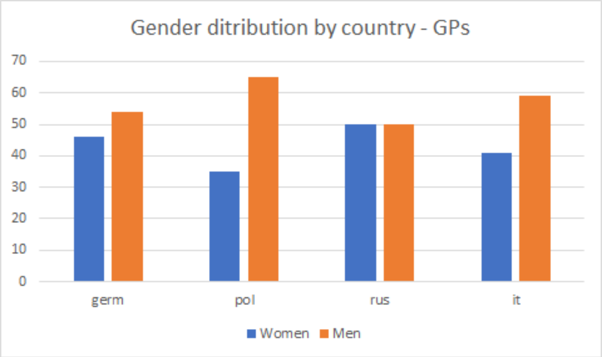

Men prevail among the interviewees, particularly in Poland and Italy, despite substantial differences between GPs and pharmacists. The pharmacists' mean age is 49.6 while doctors are a little older (52.5 years old on average).

The average number of customers / patients of pharmacists and doctors is 1,120 with high heterogeneity, in fact the number range between a minimum of 55 and a maximum of 1,600 users.

Appendix 1 – Tables by questionnaire

Pharmacists

D1. Based on your knowledge, are the following constituents of a drug are usable for the topical medication of sore throat and inflammatory/irritative conditions of the mouth such as gingivitis or stomatitis?

Values	Ketoprofene	Flurbiprofene	Ambroxolo Cloridrato	Alcool Benzilico - sodio benzoato	Benzidamina Cloridrato	Natural extracts
Yes	0.763	0.818	0.895	0.918	0.953	0.731
No	0.202	0.157	0.087	0.072	0.037	0.185
Don't know	0.035	0.025	0.017	0.01	0.01	0.085

D2. Generally speaking, which constituents do you advise for the topical treatment of sore throat and inflammatory/irritative conditions of the mouth such as gingivitis or stomatitis?

Values	Ketoprofene	Flurbiprofene	Ambroxolo Cloridrato	Alcool Benzilico - sodio benzoato	Benzidamina Cloridrato	Natural extracts
Yes	0.721	0.813	0.898	0.903	0.893	0.574
No	0.279	0.187	0.102	0.097	0.107	0.426

D3. Which of the following formulations do you recommend for the treatment of sore throat symptoms in adults? Assign an order of preference to each (1 highest, 4 lowest preference)

Preference	spray	hard candy	mouthwash	soft tab
1 Highest	0.534	0.292	0.117	0.057
2	0.182	0.272	0.362	0.185
3	0.219	0.307	0.272	0.202
4 Lowest	0.065	0.130	0.249	0.556
Avg score	1.815	2.274	2.653	3.257

D4. Which of the following constituents do you recommend for the topical treatment of sore throat symptoms in children? Assign an order of preference to each (1 highest, 3 lowest preference)

Preference	Benzidamina	Diclorobenzil	Natural extracts
1 Highest	0.369	0.444	0.187
2	0.392	0.481	0.127
3 Lowest	0.239	0.075	0.686
Avg score	1.870	1.631	2.499

D5. Which of the following formulations do you recommend for treating sore throat symptoms in children? Assign an order of preference to each (1 = most preferred formulation, 3 = least preferred formulation)

Preference	Spray	Hard candy	Soft tab
1 Highest	0.352	0.317	0.332
2	0.274	0.414	0.312
3 Lowest	0.374	0.269	0.357
Avg score	2.022	1.953	2.025

D6. For which of the following throat and oral symptoms do your clients ask for advice? (indicate in percentage)

	Ache	Itchy throat	Difficulty in swallowing	Dry throat	Mouth/throat burning	Redness of the throat	Tonsillitis
Min.	0	0	0	0	0	0	0
1st Quart.	5	0	0	0	0	5	3
Median	10	5	5	3	5	10	7
Mean	14.57	8.87	4.91	3.95	4.96	9.37	7.88
3rd Quart.	22	10	8	5	7	15	10
Max	100	70	50	30	100	40	50

	Disto rted voice	Cough	Ulcerative lesions or canker sores	Halitosis	Temperature	Symptoms due to extraction therapy	Unknown??
Min.	0	0	0	0	0	0	0
1st Quart.	0	5	0	0	0	5	0
Median	0	10	5	2	2	5	5
Mean	2.90	11.65	6.06	4.84	4.67	8.18	7.19
3rd Quart.	5	20	10	5	5	10	10
Max	30	50	50	70	70	90	100

D7. Do you recommend Benzydamine Hydrochloride (Tantum Verde)?

Values	n	n/N
Yes	308	0.768
No	93	0.232

(Administered only to pharmacists who recommend Tantum Verde)

D8. How often do you recommend Benzydamine Hydrochloride (Tantum Verde)?

Values	n	n/N	Cum-Freq
0 -| 20 %	50	0.162	0.162
20 -| 50 %	98	0.318	0.481
50 -| 70 %	130	0.422	0.903
> 70 %	30	0.097	1.000

D9. For each of the following diseases, indicate how often you recommend Benzydamine Hydrochloride (Tantum Verde) (indicate the percentage)

	Gingivitis	Stomatitis	Conservative dental therapy	Extractive dental therapy	Sore throat	Other
Min.	0	0	0	0	0	0
1st Quart.	10	10	10	10	15	0
Median	20	16	10	10	30	0
Mean	20.28	16.23	13.79	12.78	32.89	4.04
3rd Quart.	25	20	20	20	50	5
Max	100	50	50	90	100	60

D10. For which of the following symptoms reported by the customer do you recommend Benzydamine Hydrochloride (Tantum Verde)? (indicate in percentage)

	Ache	Itchy and itchy throat	Difficulty in swallowing	Dry throat	Burning of the mouth	Redness of the throat	Tonsillitis
Min.	0	0	0	0	0	0	0
1st Quart.	0	0	0	0	0	3	3.75
Median	10	5	5	2	5	10	7
Mean	12.68	8.05	5.30	3.81	5.24	9.23	8.09
3rd Quart.	20	14.25	8.25	5	10	15	10
Max	60	45	50	70	40	50	40

	Distorted voice	Cough	Ulcerative lesions or canker sores	Halitosis	Temperature	All symptoms due to extraction therapy	Gingivitis
Min.	0	0	0	0	0	0	0
1st Quart.	0	2	2	0	0	2	3
Median	0	10	5	0	0	5.5	10
Mean	2.75	10.43	8.23	4.13	2.51	9.62	9.95
3rd Quart.	5	20	10	5.25	3	10	10

Max	25	60	50	40	80	60	100

D11. Which formulation of Benzydamine Hydrochloride (Tantum Verde) do you recommend the most? (indicate the percentage)

	Mouthwash 0.15%	Spray 0.15%	Spray 0.30%
Min.	0	0	0
1st Quart.	20	20	20
Median	30	25	30
Mean	31.85	27.28	29.64
3rd Quart.	45	35	40
Max	100	80	100

D12. Do you usually provide information on the dosage and duration of treatment with Benzydamine Hydrochloride (Tantum Verde)?

Values	n	n/N
Yes	263	0.854
No	45	0.146

D13 Which are the main characteristics that make you recommend Benzydamine Hydrochloride (Tantum Verde)?

Values	Anesthetics	Analgesics	Anti-inflammatory	Antiseptics
1 Not very important	0.036	0.068	0.049	0.049
2	0.117	0.120	0.075	0.058
3	0.201	0.182	0.195	0.153
4	0.289	0.282	0.263	0.416
5 Very important	0.357	0.347	0.419	0.325

Avg score	3.815	3.721	3.929	3.909

D14. What is the percentage of consumers that ask you for advice / suggestions on Benzydamine Hydrochloride (Tantum Verde) and its use?

	Yes	No
Min.	0	0
1st Quart.	30	40
Median	50	50
Mean	48.39	51.61
3rd Quart.	60	70
Max	100	100

D15. In your experience, out of the TOTAL of customers who request / purchase Benzydamine Hydrochloride (Tantum Verde) in what percentage they choose it:

	Self-management	On medical advice	On pediatrician advice	On pharmacist advice
Min.	0	0	0	
1st Quart.	25	20	10	
Median	35	20	20	
Mean	40.03	24.24	18.98	
3rd Quart.	55	30	25	
Max	100	60	70	

Registry

Customer base

Value	n	n/N
0-|300	44	0.114
300 -| 700	274	0.712
700 - 1000	61	0.158
1000+	6	0.016

Gender

Value	n	n/N
Female	182	0.454
Male	219	0.546

Age

Min.	1st Qu.	Median	Mean	3rd Qu.	Max.
22	41	52	49.66	58	71

General practitioners

D1. According to your knowledge, are the following constituents can be used for the topical treatment of sore throat symptoms and various inflammatory / irritative conditions of the oral cavity such as gingivitis, stomatitis? (For each answer, tick Yes or No)

Values	Ketoprofene	Flurbiprofene	Ambroxolo Cloridrato	Alcool Benzilico - sodio benzoato	Benzidamina Cloridrato	Natural extracts
Yes	0.751	0.803	0.855	0.853	0.923	0.626
No	0.202	0.177	0.13	0.127	0.065	0.269
Don't know	0.047	0.02	0.015	0.02	0.012	0.105

D2. In general, which active ingredients do you prescribe / recommend for the topical treatment of sore throat symptoms and various inflammatory / irritative conditions of the oral cavity such as gingivitis, stomatitis? (For each answer, tick Yes or No)

Values	Ketoprofene	Flurbiprofene	Ambroxolo Cloridrato	Alcool Benzilico - sodio benzoato	Benzidamina Cloridrato	Natural extracts
Yes	0.698	0.761	0.813	0.835	0.808	0.491
No	0.302	0.239	0.187	0.165	0.192	0.509

D3. Based on your preference, order each of the following active ingredients for the topical treatment of sore throat symptoms and various inflammatory / irritative conditions of the oral cavity such as gingivitis, stomatitis and following extractive therapies

Value	Ketoprofene	Flurbiprofen e	Ambroxol o Cloridrato	Alcool Benzilico	Benzidamin a Cloridrato	Natural extracts	Others
1 Most pref.	0.219	0.193	0.114	0.127	0.213	0.126	0.004
2	0.232	0.168	0.212	0.225	0.099	0.044	0.027
3	0.157	0.236	0.28	0.149	0.132	0.028	0.022
4	0.157	0.204	0.172	0.256	0.165	0.036	0.013
5	0.195	0.161	0.154	0.154	0.289	0.041	0.009
6	0.040	0.035	0.053	0.076	0.094	0.692	0.031
7 Least pref	0.000	0.003	0.015	0.013	0.008	0.033	0.894
Avg score	2.995	3.085	3.260	3.365	3.530	5.031	6.664

D4. Your therapeutic approach involves (only one answer):

Values	n	n/N
Start with topical medication	95	0.401
Start with topical medication and move on to systemic	86	0.363
No preference	56	0.236

D5. Which of the following formulations do you prescribe / recommend for the treatment of sore throat symptoms in adults? (Assign an order of preference to each)

Preferenc e	Spray	Hard candy	Mouthwas h	Soft tab
1 Highest	0.566	0.287	0.105	0.042
2	0.125	0.274	0.414	0.187
3	0.242	0.379	0.224	0.155
4 Lowest	0.067	0.060	0.257	0.616
Avg score	1.810	2.212	2.633	3.344

D6. Which of the following active ingredients do you prescribe / recommend for the topical treatment of sore throat symptoms in children? (Assign an order of preference to each)

Value	Benzidamina Cloridrato	Diclorobenzil Alcool 2 mg, Sodio Benzoato 20 mg	Natural extracts
1 Most pref	0.397	0.406	0.195
2	0.401	0.441	0.155
3	0.202	0.15	0.643
4 Least pref	0.000	0.002	0.007
Avg score	1.805	1.748	2.464

D7. Which of the following formulations do you prescribe / recommend for the treatment of sore throat symptoms in children? Assign an order of preference to each (1 = most preferred formulation, 3 = least preferred formulation)

Preferenc e	Spray	Hard candy	Soft tab
1 Highest	0.384	0.266	0.349
2	0.377	0.407	0.216
3 Lowest	0.239	0.327	0.435
Avg score	1.854	2.060	2.085

D8. Do you prescribe / recommend Benzydamine hydrochloride (Tantum Verde)?

Values	n	n/N
Yes	288	0.718
No	113	0.282

(Administered only to doctors who recommend/prescribe Tantum Verde)

D9. How often do you prescribe / recommend Benzydamine hydrochloride (Tantum verde)? (only one answer)

Values	n	n/N
0 –| 20%	64	0.222
20 –| 50%	95	0.33
50 –| 70%	108	0.375
70 -|100%	21	0.073

D10. For each of the following conditions, indicate the percentage of times you prescribe / recommend Benzydamine hydrochloride (Tantum Verde)

	Gingivitis	Stomatitis	Conservative dental therapy	Extractive dental therapy	Sore throat	Other
Min.	0	0	0	0	0	0
1st Quart.	10	10	10	10	10	0
Median	20	20	15	20	20	0
Mean	19.25	16.73	16.23	15.84	27.52	4.43
3rd Quart.	25	20	20	20	40	5
Max	100	50	100	100	100	60

D11. For each of the following patient-reported symptoms, indicate the percentage of times you prescribe / recommend Benzydamine hydrochloride (Tantum Verde)

	Ache	Itchy and itchy throat	Difficulty in swallowing	Dry throat	Burning of the mouth	Redness of the throat
Min.	0	0	0	0	0	0
1st Quart.	0	0	0	0	0	0
Median	10	5	5	2	5	9

Mean	10.79	7.69	5.70	3.92	8.29	8.58
3rd Quart.	20	10	10	5	10	10
Max	90	50	50	60	50	50

	Disto rted voice	Cough	Ulcerative lesions or canker sores	Halitosis	Temperatur e	All symptoms due to extraction therapy
Min.	0	0	0	0	0	0
1st Quart.	0	0	2	0	0	5
Median	0	10	10	0	0	10
Mean	3.14	10.74	11.34	4.98	1.60	14.38
3rd Quart.	5	20	15	10	1	20
Max	75	80	100	50	20	100

D12. For each of the following formulations of Benzydamine hydrochloride (Tantum Verde), indicate the percentage of times you prescribe / recommend it

	Mouthwa sh 0.15%	Spray 0.15%	Spray 0.30%	P 3mg
Min.	0	0	0	0
1st Quart.	20	20	15	10
Median	30	20	20	10
Mean	37.40	25.07	24.61	17.15
3rd Quart.	50	30	30	25
Max	100	100	90	70

D13. Do you usually provide information on the posology and duration of treatment with Benzydamine hydrochloride (Tantum Verde)?

Values	n	n/N
Yes	228	0.719
No	89	0.281

D14. Which are the main characteristics that make you prescribe / recommend Benzydamine hydrochloride (Tantum Verde)? (indicate for all possible options the level of importance with a number from 1 to 5, e.g. 1: - Not very important - 5: Very important)

Values	Anesthetics	Analgesics	Anti-inflammatory	Antiseptics
1 Not very important	0.125	0.076	0.052	0.049
2	0.132	0.142	0.132	0.066
3	0.142	0.226	0.229	0.170
4	0.274	0.274	0.222	0.410
5 Very important	0.326	0.281	0.365	0.306
Avg score	3.545	3.542	3.715	3.858

D15. Do you prescribe / recommend Benzydamine hydrochloride (Tantum Verde) in combination with other drugs?

Values	n	n/N
Yes	203	0.705
No	85	0.295

D17 Do you prescribe / recommend Benzydamine hydrochloride (Tantum Verde) for children?

Values	n	n/N
Yes	233	0.809
No	55	0.191

Appendix 2 – Questionario utilizzato per FARMACISTI

Survey Tantum Verde per Farmacisti

1. In base alle sue conoscenze, quali dei seguenti principi attivi sono utilizzabili per il trattamento topico dei sintomi del mal di gola e delle diverse condizioni infiammatorie/irritative del cavo orale quali gengiviti, stomatiti? (Per ciascuna risposta barrare Sì oppure No)

	Sì	No	Non lo conosco
1.1 Ketoprofene			
1.2 Flurbiprofene			
1.3 Ambroxolo Cloridrato			
1.4 Alcool Benzilico - sodio benzoato			
1.5 Benzidamina Cloridrato			
1.6 Estratti naturali (es. estratto di lichene islandico (Licheni islandicum extractum), estratto di radice di altea (Althaeae radix extractum), estratto di salvia (Salviae folium extractum), Pelargonium sidoides; …)			
1.7 Altro (indicare):			

2. In generale, quali principi attivi consiglia per il trattamento topico dei sintomi del mal di gola e delle diverse condizioni infiammatorie/irritative del cavo orale quali gengiviti, stomatiti? (Per ciascuna risposta barrare Sì oppure No)

	Sì	No
2.1 Ketoprofene		
2.2 Flurbiprofene		
2.3 Ambroxolo Cloridrato		
2.4 Alcool Benzilico - sodio benzoato		
2.5 Benzidamina Cloridrato		
2.6 Estratti naturali (es. estratto di lichene islandico (Licheni islandicum extractum), estratto di radice di altea (Althaeae radix extractum), estratto di salvia (Salviae folium extractum), Pelargonium sidoides; …)		
2.7 Altro (indicare):		

3. Quali delle seguenti formulazioni raccomanda per il trattamento dei sintomi del mal di gola negli adulti? Assegni un ordine di preferenza a ciascuna

	Ordine di preferenza (1= formulazione maggiormente preferita, 4=formulazione meno preferita)
3.1 Spray	
3.2 Caramelle	
3.3 Collutorio	

3.4 Pastiglie gommose	

4. Quale dei seguenti principi attivi raccomanda per il trattamento topico dei sintomi del mal di gola nei bambini? Assegni un ordine di preferenza a ciascuno

	Ordine di preferenza (1= principio maggiormente preferito, 3=principio meno preferito)
4.1 Benzidamina Cloridrato	
4.2 Diclorobenzil Alcool 2 mg, Sodio Benzoato 20 mg	
4.3 Estratti naturali (es. estratto di lichene islandico (Licheni islandicum extractum), estratto di radice di altea (Althaeae radix extractum), estratto di salvia (Salviae folium extractum), Pelargonium sidoides; …)	

5. Quali delle seguenti formulazioni raccomanda per il trattamento dei sintomi del mal di gola nei bambini? Assegni un ordine di preferenza a ciascuna (1= formulazione maggiormente preferita, 3=formulazione meno preferita)

	Ordine di preferenza (1= formulazione maggiormente preferita, 3=formulazione meno preferita)
5.1 Spray	
5.2 Caramelle dure?	
5.3 Pastiglie gommose	

6. Per quali delle seguenti sintomatologie della gola e del cavo orale i clienti le chiedono consiglio ? *(indicare in percentuale)*

6.1 Dolore	
6.2 Pizzicore e prurito alla gola	
6.3 Difficoltà nella deglutizione	
6.4 Secchezza della gola	
6.5 Bruciore della bocca o della gola	
6.6 Arrossamento della gola	
6.7 Gonfiore delle tonsille	
6.8 Voce alterata – rauca/abbassata	
6.9 Tosse	
6.10 Lesioni ulcerose o afte	
6.11 Alitosi	
6.12 Febbre	
6.13 Tutti i sintomi dovuti alla terapia estrattiva	

7. Consiglia Benzidamina Cloroidrato (Tantum Verde)?

7.1 Sì	
7.2 No	

Le domande successive vanno somministrate solo se l'intervistato risponde Sì alla 7

8. Con quale frequenza consiglia Benzidamina Cloroidrato (Tantum Verde)?

8.1 0 – 20%	
8.2 21 – 50%	
8.3 51 – 70%	
8.4 > 70%	

9. Per ciascuna delle seguenti patologie, indichi la frequenza con la quale consiglia Benzidamina Cloroidrato (Tantum Verde) *(indicare la percentuale)*

9.1 Gengiviti	
9,2 Stomatiti	
9.3 Terapia dentaria conservativa	
9.4 Terapia dentaria estrattiva	
9.5 Mal di gola	
9.6 Altro	

10. Per quali delle seguenti sintomatologie riferite dal cliente consiglia Benzidamina Cloroidrato (Tantum Verde)? *(indicare in percentuale)*

10.1 Dolore	
10.2 Pizzicore e prurito alla gola	
10.3 Difficoltà nella deglutizione	
10.4 Secchezza della gola	
10.5 Bruciore della bocca o della gola	
10.6 Arrossamento della gola	
10.7 Gonfiore delle tonsille	
10.8 Voce alterata – rauca/abbassata	
10.9 Tosse	
10.10 Lesioni ulcerose o afte	
10.11 Alitosi	
10.12 Febbre	
10.13 Tutti i sintomi dovuti alla terapia estrattiva	

11. Quale formulazione di Benzidamina Cloroidrato (Tantum Verde) consiglia maggiormente? *(indicare la percentuale)*

11.1 Collutorio 0.15%	
11.2 Spray 0.15%	
11.3 Spray 0.30%	
11.4 P 3mg	

12. Solitamente, fornisce indicazioni sulla posologia e sulla durata del trattamento con Benzidamina Cloroidrato (Tantum Verde)?

12.1Sì	
12.2No	

13. Per quali principali caratteristiche del farmaco consiglia Benzidamina Cloroidrato (Tantum Verde)?

(indicare per tutte le possibili opzioni il livello di importanza con un numero da 1 a 5; es: 1: poco importante – 5: molto importante)	
13.1 Anestetiche	
13.2 Analgesiche	
13.3 Antifiammatorie	
13.4 Antisettiche	

14 I consumatori Le chiedono consigli/suggerimenti su Benzidamina Cloroidrato (Tantum Verde) e il suo impiego? *(indicare la percentuale)*

14.1Sì	
14.2No	

15. Nella sua esperienza, sul TOTALE dei clienti che richiedono/acquistono Benzidamina Cloroidrato (Tantum Verde) in che percentuale lo scelgono:

	Indicare la percentuale (il totale deve sommare a 100)
15.1 In autonomia (self-management)	
15.2 Su indicazione/prescrizione MMG	
15.3 Su indicazione/prescrizione del pediatra	
15.4 Su suo consiglio di farmacista	

INFORMAZIONI ANAGRAFICHE

A1: Sesso____________________________

A2: Anno di nascita _______________________________

A3: In quale anno si è iscritto all'ordine __________________________

A4: Indichi il luogo dove opera (municipio, quartiere) _________________________________

A5: Indichi il bacino di utenza potenziale _________________________

Survey Tantum Verde per Medici

1. In base alle sue conoscenze, quali dei seguenti principi attivi sono utilizzabili per il trattamento topico dei sintomi del mal di gola e delle diverse condizioni infiammatorie/irritative del cavo orale quali gengiviti, stomatiti? (Per ciascuna risposta barrare Sì oppure No)

	Sì	No	Non lo conosco
1.1 Ketoprofene			
1.2 Flurbiprofene			
1.3 Ambroxolo Cloridrato			
1.4 Alcool Benzilico - sodio benzoato			
1.5 Benzidamina Cloridrato			
1.6 Estratti naturali (es. estratto di lichene islandico (Licheni islandicum extractum), estratto di radice di altea (Althaeae radix extractum), estratto di salvia (Salviae folium extractum), Pelargonium sidoides; …)			
1.7 Altro (indicare):			

2. In generale, quali principi attivi prescrive/consiglia per il trattamento topico dei sintomi del mal di gola e delle diverse condizioni infiammatorie/irritative del cavo orale quali gengiviti, stomatiti? (Per ciascuna risposta barrare Sì oppure No)

	Sì	No
2.1 Ketoprofene		
2.2 Flurbiprofene		
2.3 Ambroxolo Cloridrato		
2.4 Alcool Benzilico - sodio benzoato		
2.5 Benzidamina Cloridrato		
2.6 Estratti naturali (es. estratto di lichene islandico (Licheni islandicum extractum), estratto di radice di altea (Althaeae radix extractum), estratto di salvia (Salviae folium extractum), Pelargonium sidoides; …)		
2.7 Altro (indicare):		

3. Assegni un ordine di preferenza a ciascuno dei seguenti principi attivi per il trattamento topico dei sintomi del mal di gola e delle diverse condizioni infiammatorie/irritative del cavo orale quali gengiviti, stomatiti e a seguito di terapie estrattive

	Ordine di preferenza (1= principio attivo maggiormente preferito, 7= principio attivo meno preferito)
3.1 Ketoprofene	

3.2 Flurbiprofene	
3.3 Ambroxolo Cloridrato	
3.4 Alcool Benzilico - sodio benzoato	
3.5 Benzidamina Cloridrato	
3.6 Estratti naturali (es. estratto di lichene islandico (Licheni islandicum extractum), estratto di radice di altea (Althaeae radix extractum), estratto di salvia (Salviae folium extractum), Pelargonium sidoides; …)	
3.7 Altro (indicare):	

4. Il suo approccio terapeutico prevede (una sola risposta):

4.1 Iniziare con farmaco topico	
4.2 Iniziare con farmaco topico e passare al sistemico	
4.3 Nessuna preferenza	

5. Quali delle seguenti formulazioni prescrive/raccomanda per il trattamento dei sintomi del mal di gola negli adulti? Assegni un ordine di preferenza a ciascuna

	Ordine di preferenza (1= formulazione maggiormente preferita, 4=formulazione meno preferita)
5.1 Spray	
5.2 Caramelle	
5.3 Collutorio	
5.4 Pastiglie gommose	

6 Quale dei seguenti principi attivi prescrive/raccomanda per il trattamento topico dei sintomi del mal di gola nei bambini? Assegni un ordine di preferenza a ciascuno

	Ordine di preferenza (1= principio maggiormente preferito, 3=principio meno preferito)
6.1 Benzidamina Cloridrato	
6.2 Diclorobenzil Alcool 2 mg, Sodio Benzoato 20 mg	
6.3 Estratti naturali (es. estratto di lichene islandico (Licheni islandicum extractum), estratto di radice di altea (Althaeae radix extractum), estratto di salvia (Salviae folium extractum), Pelargonium sidoides; …)	

7. Quali delle seguenti formulazioni prescrive/raccomanda per il trattamento dei sintomi del mal di gola nei bambini? Assegni un ordine di preferenza a ciascuna (1= formulazione maggiormente preferita, 3=formulazione meno preferita)

	Ordine di preferenza (1= formulazione maggiormente preferita, 3=formulazione meno preferita)
7.1 Spray	
7.2 Caramelle dure?	
7.3 Pastiglie gommose	

8. Prescrive/consiglia Benzidamina cloroidrato (Tantum Verde)?

8.1 Sì	
8.2 No	

<u>Le domande successive vanno somministrate solo se l'intervistato risponde Sì alla 8</u>

9. Con quale frequenza prescrive/consiglia Benzidamina cloroidrato (Tantum verde)? (una sola risposta)

9.1 0 – 20%	
9.2 21 – 50%	
9.3 51 – 70%	
9.4 > 70%	

10. Per ciascuna delle seguenti patologie, indichi la percentuale delle volte in cui prescrive/consiglia Benzidamina cloroidrato (Tantum Verde)

	Indicare la percentuale
10.1 Gengiviti	
10.2 Stomatiti	
10.3 Terapia dentaria conservativa	
10.4 Terapia dentaria estrattiva	
10.5 Mal di gola	
10.6 Altro (indicare):	

11. Per ciascuna delle seguenti sintomatologie riferite dal paziente, indichi la percentuale delle volte in cui prescrive/consiglia Benzidamina cloroidrato (Tantum Verde)

	Indicare la percentuale
11.1 Dolore	
11.2 Pizzicore e prurito alla gola	
11.3 Difficoltà nella deglutizione	
11.4 Secchezza della gola	
11.5 Bruciore della bocca o della gola	
11.6 Arrossamento della gola	
11.7 Gonfiore delle tonsille	
11.8 Voce alterata – rauca/abbassata	
11.9 Tosse	
11.10 Lesioni ulcerose o afte	
11.11 Alitosi	

11.12 Febbre	
11.13 Tutti i sintomi dovuti alla terapia estrattiva	

12. Per ciascuna delle seguenti formulazioni di Benzidamina cloroidrato (Tantum Verde), indichi la percentuale delle volte in cui la prescrive/consiglia.

	Indicare la percentuale
12.1 Colluttorio 0.15%	
12.2 Spray 0.15%	
12.3 Spray 0.30%	
12.4 P 3mg	

13. Solitamente, fornisce indicazioni sulla posologia e sulla durata del trattamento con Benzidamina cloroidrato (Tantum Verde)?

13.1 Sì	
13.2 No	

14. Per quali principali caratteristiche del farmaco prescrive/consiglia Benzidamina cloroidrato (Tantum Verde)? *(indicare per tutte le possibili opzioni il livello di importanza con un numero da 1 a 5, es. 1:- poco importante – 5: molto importante)*

Caratteristica	Livello di importanza (1: poco importante – 5 molto importante)
14.1 Anestetiche	
14.2 Analgesiche	
14.3 Antinfiammatorie	
14.4 Antisettiche	

15. Prescrive/consiglia Benzidamina cloroidrato (Tantum Verde) in associazione con altri farmaci?

15.1 Sì	
15.2 No	

16. Se sì, quali altri farmaci prescrive/consiglia in associazione a Benzidamina cloroidrato (Tantum Verde)? (E' ammessa più di una risposta)

16.1 Antinfiammatori	
16.2 Antibiotici	
16.3 Analgesici	
16.4 Altro (indicare):	

17. Prescrive/consiglia Benzidamina cloroidrato (Tantum Verde) per i bambini?

17.1 Sì	
17.2 No	

INFORMAZIONI ANAGRAFICHE

A1: Sesso____________________________

A2: Anno di nascita _______________________________

A3: In quale anno si è iscritto all'ordine __________________________

A4: Indichi il luogo dove opera (municipio, quartiere) _________________________________

A5: Indichi il numero di pazienti che assiste_________________________
